# CD137 Signaling Modulates Vein Graft Atherosclerosis by Driving T-Cell Activation and Regulating Intraplaque Angiogenesis

**DOI:** 10.1016/j.jacbts.2025.101323

**Published:** 2025-07-29

**Authors:** Alwin de Jong, Thijs J. Sluiter, Hendrika A.B. Peters, Alec Lamens, J. Wouter Jukema, Ramon Arens, Paul.H.A. Quax, Margreet R. de Vries

**Affiliations:** a Department of Surgery, Leiden University Medical Center, Leiden, the Netherlands; b Einthoven Laboratory for Experimental Vascular Medicine, Leiden University Medical Center, Leiden, the Netherlands; c Department of Cardiology, Leiden University Medical Center, Leiden, the Netherlands; d Netherlands Heart Institute, Utrecht, the Netherlands; e Department of Immunology, Leiden University Medical Center, Leiden, the Netherlands

**Keywords:** CD137-CD137L signaling, costimulation, intraplaque angiogenesis, T-cells, vein graft atherosclerosis

## Abstract

Atherosclerotic vein graft failure still presents a major problem. T-cells have been identified as one of the most abundant immune cell subset in atherosclerotic plaques. Their role, however, remains only partly understood. Using a murine vein graft model for advanced, unstable atherosclerotic lesions, we find that T-cells accumulate over time in atherosclerotic vein grafts, and appear to be activated rapidly after engraftment, demonstrated by increased expression of CD137 on plaque T-cells. Targeting of CD137 affects intraplaque angiogenesis and plaque growth, which renders CD137 a promising target for early immunomodulation to reduce atherosclerotic vein graft failure.

## Highlights

• CD8^+^, and to a lesser extent CD4^+^, T-cells accumulated over time in murine unstable, atherosclerotic vein graft lesions, while their activation occurs rapidly after surgery.
• CD137 signaling regulated predominantly CD8^+^ T-cell activation, effector-memory differentiation, and IFN-γ expression.
• Early immunomodulation (1 day after surgery) through agonistic CD137 treatment improved vein graft remodeling and diminished intraplaque angiogenesis, whereas silencing of CD137 signaling by antagonistic antibodies aggravated atherosclerotic lesion development.

## Visual Abstract

**Figure:**
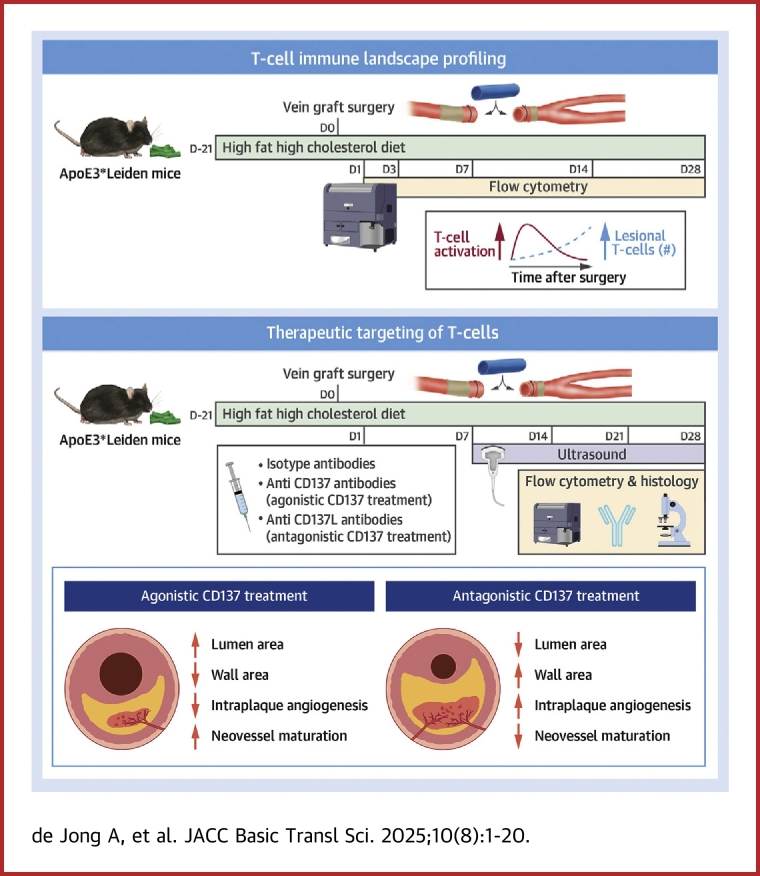

Vascular inflammation is a fundamental driver of atherosclerosis, characterized by macrophages infiltrating the vessel wall to clear oxidized low-density lipoprotein.^1^^,^^2^ Recent single-cell technologies, however, have unveiled a significant presence of T-cells within atherosclerotic plaques.^3^, ^4^, ^5^ These T-cells frequently display an activated phenotype, suggesting that T-cells actively contribute to atherosclerotic plaque fate.^6^^,^^7^ Their causality and role in atherogenesis, however, remains to be fully understood caused by large variety of T-cell subsets and opposing functions.

Multiple T-cell subsets have been identified in atherosclerotic plaques including naïve, central memory, and effector-memory T-cells.^6^^,^^8^ Naïve T-cells are precursors for all effector and memory T-cell subsets. Following T-cell receptor triggering and costimulation in lymphoid organs, these cells proliferate and differentiate into effector T-cells and memory T-cell subsets. Central memory T-cells are characterized by their presence in blood, lymphoid organs and sustained response, whereas effector-memory T-cells are characterized by rapid effector function, production of various (inflammatory) cytokines, and their rapid infiltration into inflamed tissues.^9^ It has been described that increasing density of effector T-cells associates with atherosclerotic plaque instability.^3^^,^^4^^,^^6^^,^^7^ Moreover, oligoclonal expansion of effector CD4^+^ and CD8^+^ T-cells has been observed, including terminally differentiated effector-memory T-cells within atherosclerotic lesions, suggestive of active contribution of T-cells to plaque inflammation and lesion development.^6^^,^^10^^,^^11^ Preclinical studies investigating the causal role of T-cells in murine atherosclerosis have mainly been conducted via either CD4^12^, ^13^, ^14^ or CD8^14^^,^^15^ antibody depletion or adoptive T-cell transfer,^16^^,^^17^ yielding seemingly conflicting results. For example, cytotoxic CD8^+^ T-cells have been described to be proatherogenic,^15^ but have also been observed to inhibit atherogenesis, when guided via vaccination.^18^^,^^19^ Therefore, therapeutic targeting of specific T-cell subsets might aid in eliciting atheroprotective rather than atherogenic T-cell responses to reduce disease burden.

Costimulatory and inhibitory molecules expressed by antigen-presenting cells, together with cytokines, regulate T-cell activation and subsequent differentiation into specific T-cell subsets.^8^ Multiple costimulatory and inhibitory molecules have been described to critically influence cardiovascular diseases such as CD40-CD40L^20^^,^^21^ and CD70/80/86.^8^^,^^22^ CD137, also known as 4-1BB, is a costimulatory molecule that is predominantly expressed on activated CD8^+^ T-cells, and agonistic stimulation of CD137 has demonstrated efficacy as an antitumor therapy.^23^

Cross-linking of CD137 and the T-cell receptor on activation T-cells can deliver costimulatory signals that result in T-cell proliferation, survival, memory formation, as well as increased cytotoxicity and cytokine production. CD137 is expressed in human atherosclerotic lesions, but not in normal arteries. Additionally, CD137 mRNA levels are approximately 10-fold higher in atherosclerosis-prone mice compared with wildtype. Furthermore, activation of CD137 increased CD8^+^ T-cell infiltration and resulted in accelerated (early) plaque development in mice. Moreover, genetic silencing reduced atherogenesis, suggesting a proatherosclerotic role for CD137. Recent evidence, however, demonstrated an inverse correlation between CD137 mRNA expression and cerebrovascular or peripheral vascular events.^24^ Moreover, CD137 protein expression was reduced in symptomatic plaques compared with nonsymptomatic ones. Altogether, this demonstrates the complexity of CD137-CD137L signaling and underscores the need for further investigation vascular pathophysiology.^25^

We have previously demonstrated that activated CD4^+^ and CD8^+^ T-cells are also found in (murine) atherosclerotic vein grafts, which are reminiscent of advanced, unstable human atherosclerotic plaques.^5^ Moreover, these atherosclerotic vein grafts exhibit intraplaque angiogenesis and intraplaque hemorrhage, which are not observed in murine models for naïve atherosclerosis.^26^^,^^27^ The effects of T-cells on intraplaque angiogenesis and formation of unstable atherosclerotic lesions remains largely unknown. In murine vein grafts, an abundance of CD8^+^ T-cells was found in comparison to other organs, while transcriptomics indicated T-cell receptor signaling as significantly up-regulated after surgery. Interestingly, antibody-mediated depletion of CD8^+^, but not CD4^+^, T-cells in vein-grafted mice resulted in reduced vein graft patency, suggesting an atheroprotective role for CD8^+^ T-cells.^5^

In our current study, we assessed the T-cell immune landscape at various timepoints reflecting early, mid, and late stages of lesion development, to delineate the role of T-cells in unstable atherosclerotic vein grafts. We observed a progressive increase in the total number of T-cells over time and identified CD137 as a key costimulatory molecule expressed mainly on CD8^+^ T-cells in the initial phase of vascular remodeling following venous bypass surgery. Interrogating the role of CD137-CD137L signaling by therapeutic targeting through administration of agonistic as well as antagonistic antibodies in vivo revealed a prominent effect of this costimulatory receptor-ligand pair on intraplaque angiogenesis and formation of unstable atherosclerotic vein graft lesions.

## Methods

### Study approval and mice

This study was performed in compliance with the Dutch government guidelines and Directive 2010/63/EU of the European Parliament. The institutional committee of the Leiden University Medical Centre approved all the animal experiments licensed under project numbers (11045, 116002016645). Male C57BL/6 ApoE3∗Leiden+/− (ApoE3∗Leiden) mice were fed with a Western-type diet containing 1.0% cholesterol and 0.5% cholate (HFD) (Sniff Spezialdiäten, GMBH, Soest, Germany) to induce hypercholesterolemia.^28^ Male mice were used as recipients while both male and female mice were used as donor. Mice were obtained from an in-house breeding colony and received food and water ad libitum during the entire experiment.

### Experimental design

The vein graft procedure involves the isolation of caval veins, which were obtained from male and female donor littermates of the same age (10-16 weeks) as the ApoE3∗Leiden^+/−^ recipient mice. Both donor and recipient mice were anesthetized by intraperitoneal injection consisting of a combination of midazolam (5 mg/kg, Roche), medetomidine (0.5 mg/kg, Orion), and fentanyl (0.05 mg/kg, Janssen). Response to toe pinching of the mice as well as breathing frequency was used to assess anesthesia adequacy. Vein graft surgery was performed as described previously^29^ by interpositioning the caval vein into the arterial circulation of a recipient at the site of the right common carotid artery. After surgery and on indication, buprenorphine (0.1 mg/kg, Merck Sharp & Dohme Animal Health) was given as an analgesic. Before surgery, mice were randomized into different groups based on their plasma cholesterol values, measured according to manufacturer’s protocol (Roche, 1489437) before surgery. Mice with plasma cholesterol values of <10 or >35 mmol/L were excluded from the study and used as donor mice. At sacrifice, vein-grafted mice were anesthetized (using the previously described midazolam/medetomidine/fentanyl combination) and sacrificed through collection of whole blood via the orbital sinus. Following exsanguination, the abdomen was opened and 5-mL phosphate-buffered saline (Braun) was used to perfuse the circulation from the left ventricle. Thereafter, tissue (including the vein graft) was either harvested and processed for flow cytometric analysis or 4% paraformaldehyde was used for perfusion to fixate tissue to be used for immunohistochemistry. A priori power calculations (using a power of 0.95 and effect size of 0.89, based on previous studies) were performed to determine number of animals needed per group (n = 8). Due to nonsurvival and occlusion as a result of thrombosis (which were excluded from analysis), we included extra animal(s) to compensate for this loss, which can result in intraexperiment group size differences. Please find below the antibodies used for pharmacological targeting of CD137 and CD137L.

### Noninvasive ultrasound

The animals were anesthetized with isoflurane and placed on the mouse imaging platform of the Vevo LAZR-X system (VisualSonics, FUJIFILM), where temperature, heart rate, and respiration rate were monitored in real-time. During the ultrasound acquisitions, anesthesia was maintained using a vaporized isoflurane gas system (1 L/min of oxygen, 0.3 L/min air, and 2.5% isoflurane). The concentration of isoflurane was adjusted according to the pedal reflex and respiration rate to ensure adequate anesthesia. The region ranging from the salivary gland to the sternum was shaved and covered with ultrasound gel. Ultrasound measurements of vascular remodeling were performed with the mx550 transducer. Short-axis scanning 3-dimensional (3D) B-mode, and 3D color Doppler images were acquired weekly. Image visualization, reconstruction, and processing were realized with VevoLAB 3.2.6 software (FUJIFILM, VisualSonics), as described previously.^30^ Detailed analyses were performed using a 3D, short-axis ultrasound method with which lumen, vessel wall, and total vessel (lumen + vessel wall) area were calculated based on the mean of 5 measurement sites (caudal, medial, and cranial).

### Flow cytometry

The isolated vein grafts were processed for single-cell flow cytometric analysis. As a whole, the vein grafts were minced in small pieces and strained by a 30-μm strainer (Greiner), flushed with phosphate-buffered saline supplemented with 5-μm EDTA and 1% fetal calf serum (Flow buffer) to obtain single-cell suspensions. After 2 washing steps, the pellet was resuspended in anti-CD16/32 (BD Biosciences) and incubated for 20 minutes. The antibodies used for flow cytometric analysis are listed in Table 1. The antibody mix was incubated for 30 minutes, and after 2 washing steps with flow buffer, analyzed by the BD Fortessa or Cytek Aurora. Flow cytometric analysis was performed by Flowjo VX. The polarization of T-cells was assessed by injecting 0.25 mg Brefeldin A^31^ (Biolegend) in the tail veins of vein-grafted ApoE3∗Leiden mice. After 6 hours, the accelerated atherosclerotic lesion, spleen, blood, and draining and nondraining lymph nodes were harvested. Intracellular staining was performed according to BD Biosciences protocol.

**Table 1:** Monoclonal Used for Flow Cytometry and Immune Histochemistry

Marker	Fluorochrome	Dilution	Clone	Supplier	Application
Viability	Zombie-UV	200	—	Biolegend	Flow
CD137	BV421	200	1AH2	BD Biosciences	Flow
CD25	APC	300	3C7	Biolegend	Flow
CD3	BV421	200	145-2C11	BD Biosciences	Flow
CD3	BV510	100	145-2C11	BD Biosciences	Flow
CD3	BV650	100	145-2C11	BD Biosciences	Flow
CD3	BV711	200	145-2C11	BD Biosciences	Flow
CD3	PE	100	145-2C11	Biolegend	Flow
CD3	APC	100	145-2C11	Biolegend	Flow
CD3	AF647	100	145-2C11	Biolegend	Flow
CD3	APC Fire 750	100	145-2C11	Biolegend	Flow
CD3	FITC	300	145-2C11	Biolegend	Flow
CD4	Pe-Cy7	800	RM4-5	BD Biosciences	Flow
CD43gl	PE-dazzle	200	1B11	Biolegend	Flow
CD44	BV711	600	IM7	Biolegend	Flow
CD45.2	BV785	100	104	Biolegend	Flow
CD45.2	PE Dazzle	200	104	Biolegend	Flow
CD62L	APC Fire750	400	MEL-14	Biolegend	Flow
CD69	FITC	100	H1.2F3	Biolegend	Flow
CD8	BV605	200	53.6-7	Biolegend	Flow
CD8	PE-cy5	600	53.6-7	Biolegend	Flow
CD8	APC R700	200	53.6-7	BD Biosciences	Flow
IFN-γ	BV650	200	XMG1,2	BD Biosciences	Flow
IL-17A	AF647	200	TC11-18H10	BD Biosciences	Flow
Il-4	PE	400	11B11	BD Biosciences	Flow
KLRG1	PE-cy5	200	2F1	Thermo Fischer	Flow
PD-1	APC R700	400	RMP1-30	BD Biosciences	Flow
CD107b	Unconjugated	100	M3/4	BD Biosciences	IHC-P
ACTA2	Unconjugated	1,000	1A4	Dako	IHC-P
ACTA2	Alexa fluor 555	800	EPR5368	Abcam	IHC-P (F)
CXCL10 (IP10)	Unconjugated	200	10H11L3	Thermo Fischer	IHC-P (F)
Donkey antirabbit	Alexa fluor 647	1,000	—	Thermo Fischer	IHC-P (F)
Donkey antirat	Alexa Fluor 555	1,000	—	Thermo Fischer	IHC-P (F)
CD31	Unconjugated	3,000	RM1006	Abcam	IHC-P (F)
CD137	Unconjugated	200	BVR051F	Abcam	IHC-P(F)
CD3	Unconjugated	100	SP7	Abcam	IHC-P(F)
CXCL10	Unconjugated	200	10H11L3	Thermo Fischer	IHC-P(F)
CD4	Alexa fluor 555	100	EPR19514	Abcam	IHC-P(F)
CD8	Alexa fluor 647	100	ERP21769	Abcam	IHC-P(F)
CD137	Unconjugated	200	EPR23218-111	Abcam	IHC-P
CD137	Unconjugated	100	Ab197942	Abcam	IHC-P

AF= Alexa Fluor; APC = antigen-presenting cells; BV = Brilliant Violet; IHC-F= immunohistochemistry-frozen; IHC-P immunohistochemistry-paraffin.

### Morphometric and compositional analysis

Atherosclerotic vein graft lesions were sectioned in sequential 5-μm-thick cross-sections made throughout the entire vein graft. The plastic cuff served as the starting point for mounting sections onto glass slides. The total vein was analyzed by a minimum of 6 equally spaced sections. Each staining was performed on comparable regions of the vein grafts. Movat Pentachrome staining was used for morphometric analysis of vein graft atherosclerotic lesions. From this staining, the lumen, intima, and total atherosclerotic area were measured. Sirius red staining (Klinipath 80,115) was used to quantify the amount of collagen I/III present in the vessel wall. Primary antibodies (see Table 2) binding to vascular smooth muscle cells and the macrophages were visualized by the DAB substrate complex. These slides were scanned using the Panoramic Scan II (3D Histech). Immunofluorescence was used to quantify the number of neovessels and their maturation status. Maturation was assessed by ACTA2 coverage of CD31^+^ neovessels and scored by a blinded observer. When <6 sections were available for analysis (eg, because of out-of-focus scans or damage to sections during immunohistochemistry), mice were not included in the analysis. Anonymous human coronary artery vein graft specimens (n = 4) were obtained at the LUMC in accordance with guidelines set out by the “Code for Proper Secondary Use of Human Tissue” of the Dutch Federation of Biomedical Scientific Societies (Federa) and conform the principles outlined in the Declaration of Helsinki. These human as well as murine vein grafts were stained for CD3 and CD137, secondary Alexa Fluor 555 and Alexa Fluor 647 were used to visualize primary antibody binding on human vein grafts. Immunofluorescent stainings were scanned using the Zeiss Axioscan V2 (Zeiss) and representative images were taken by confocal microscopy (Zeis LSM710). Analysis was performed using ImageJ (FIJI).

**Table 2:** Pharmacological Targeting of CD137 and CD137L

Name	Clone	Catalog	Lot	Manufacturer	**μ**g/mice	Administration
Anti-CD137 (agonistic treatment)	3H3	BP0239	661217D1	Bio X Cell	200	1× s.c. (at t = 1d)
Anti-CD137L (antagonistic treatment)	TKS-1	BE0110	664520F1	Bio X Cell	200	twice/week s.c. (starting at t = 1d)
Isotype control	LTF-2	BP0090	707119S1	Bio X Cell	200	twice/week s.c. (starting at t = 1d)

s.c. = subcutaneous.

### IFN-γ measurements

Peripheral blood mononuclear cells were isolated from blood obtained from healthy volunteers by density centrifugation. The ethics committee of the LUMC approved the study, which was conducted in accordance with Declaration of Helsinki and written consent was given by the healthy volunteers. The CD14^+^ (130-050-201, Miltenyi Biotec) monocytes were isolated with beads according to the manufacturer’s protocol. After isolation, the monocytes were frozen and stored in liquid nitrogen before dendritic cells (DCs) differentiation. The CD14^−^ fraction containing the CD3^+^ T-cells was stimulated with anti-CD3 (OKT3, Biolegend) in an AIM-V medium (Invitrogen). On t = 1d and t = 3d, the cells were washed twice and resuspended at 1.0 × 10^6^ cells/mL. Interleukin-2 (Proleukin) 1,000 U/mL and interleukin-15 100 U/mL were added to the culture medium. Depending on the growth rate, cells were split in multiple culture flasks. At t = 11d, the cells were sorted (BD Aria) in a CD4^+^ population and a CD8^+^ population and restimulated with OKT3 when proliferation resided. The monocytes were thawed and differentiated with GM-CSF (20 ng/mL) toward DCs for 6 days and subsequently maturated with LPS. The mature DCs were primed either with the human CD4^+^ peptide pool (CEFX, JPT) or the CD8^+^ (CEFX, JPT) peptide pool for 6 hours before the DC T-cell coculture. Per well, 10.000 DCs with 50.000 T-cells were added in the presence of agonistic CD137 antibodies (Recombinant Human 4-1BBL, 750002, Biolegend). The IFN-γ ELISpot (Mabtech) with MSIP plates (Millipore) were used according to the manufacturer’s protocols. The IFN-γ in murine plasma concentrations were measured by using the BD Biosciences detection kit (555138) according to the manufacturer’s instructions.

### Aortic ring assay

The aortic ring assay was performed as described previously.^27^^,^^32^ Three C57Bl6/J mice, age 4 to 8 weeks, were anesthetized and the aorta was dissected. Each aorta was cut in 1-mm rings, and serum starved in Gibco Optimem GlutaMAX (51985034, Thermo Fisher Scientific) overnight at 37 °C and 5% CO_2_. On the next day, each ring was mounted in a well of a 96-well plate in 70 μL of 1 mg/mL acid-solubilized collagen type-1 (1117979001, Roche Diagnostics) in DMEM (12634010, Thermo Fisher Scientific). After collagen polymerization, Optimem supplemented with 10% FCS, 1% glutamine, and 25 ng/mL VEGF (293-VE, R&D systems) was added to the aortic rings. Isotype and anti-CD137 (both 100 ng/mL) were added to assess the direct effect of anti-CD137 on sprouting. To assess the effect of CD8^+^ T-cells, these cells were isolated from the spleen of a C57Bl6/J mouse, age >8 weeks, using the CD8 enrichment kit (558471, BD Biosciences). These cells were cultured in IMDM and stimulated with anti-CD3 (1 μg/mL, 553057, BD Biosciences), anti-CD28 (2 μg/mL, 553294, BD Biosciences), and/or anti-CD137 (2 μg/mL) for 24 hours. Thereafter, cells were counted and 10.000 cells (without the anti-CD3, -CD28, and/or CD137 antibodies) were added to each well. 30 replicates (wells) were used for each condition. The rings were cultured for 6 days and photographed using an Axiovert 40c microscope. The number of sprouts were counted manually by a blinded, experienced observer (T.J.S.).

### Statistical analysis

Comparisons among >2 independent groups were done using 1- or 2-way analysis of variance or Kruskal-Wallis test with Dunnett's or Dunn's post hoc test, respectively, for multiple pairwise comparisons to a control. Agonistic and antagonistic CD137 treatment were only compared with isotype-control. Variables measured over time (longitudinal) or with repeated measurements from the same animal were analyzed using 2-way repeated measures analysis of variance or mixed-effects analysis with correction for multiple testing to a control (Dunnett test). Statistical analyses were performed with GraphPad Prism version 9.3.1 (GraphPad Software), and a *P* value <0.05 was considered statistically significant.

## Results

### Predominantly CD8^+^ T-Cells Accumulate in Atherosclerotic Vein Graft Lesions During Plaque Development

**Figure 1:**
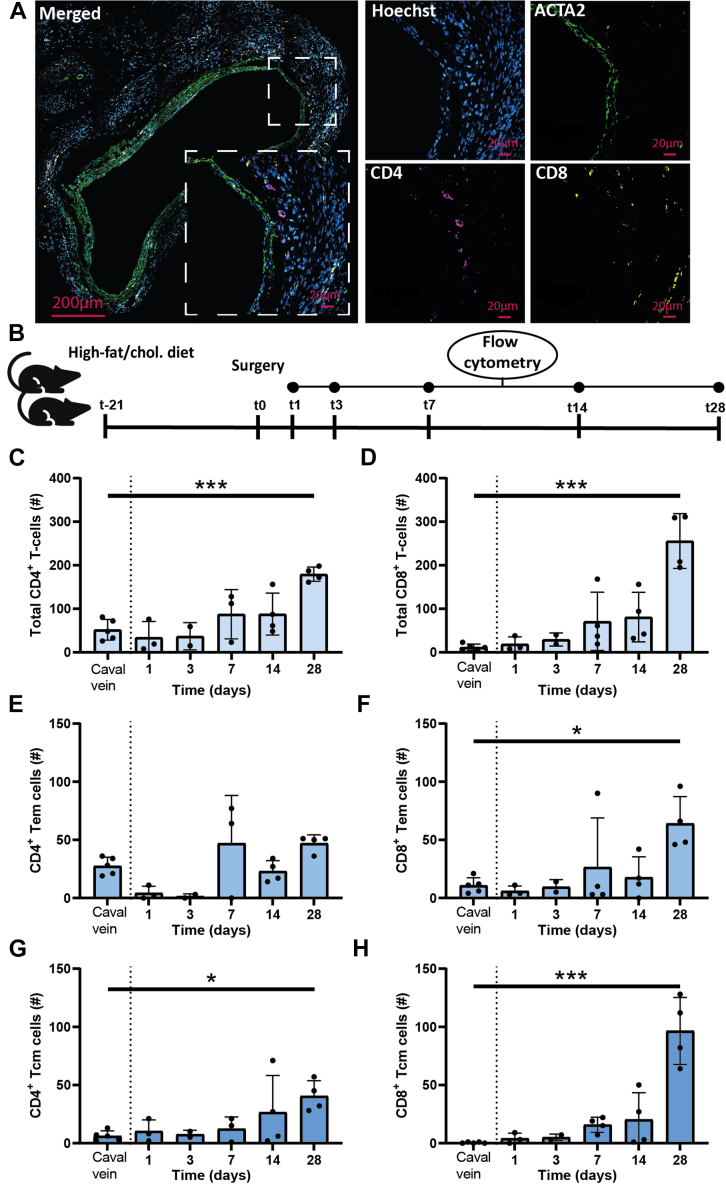
Predominantly CD8^+^, and to a Lesser Extent CD4^+^ T-Cells, Accumulate in the Vein Graft Wall During Vein Graft Remodeling Atherosclerotic vein graft lesions stained for CD4, CD8, Hoechst, and ACTA2 (A). Experimental setup: caval veins and vein grafts of high-fat/cholesterol-fed ApoE3∗Leiden mice (n = 3-4 per timepoint) were harvested at different timepoints and processed for flow cytometry (B). Quantification of flow cytometric analysis: absolute number of total CD4^+^ (C) and CD8^+^ (D) T-cell population as well as absolute number of effector-memory T-cells (Tem) (CD62L^−^ CD44^+^) (E,F) and central-memory T-cells (Tcm) (CD62L^+^ CD44^+^) (G,H). Data are represented as mean ± SD and were tested using 1-way analysis of variance with Dunnett post hoc test; significance is indicated for relevant comparisons and ∗*P <* 0.05, ∗∗*P <* 0.01, and ∗∗∗*P <* 0.001.

Both CD4^+^ and CD8^+^ T-cells are found in atherosclerotic vein graft lesions, where they reside in all layers of the vessel wall (Figure 1A). To characterize these lesional T-cells and examine the immune landscape of T-cells during different stages of lesion development, single-cell suspensions from atherosclerotic vein grafts were obtained at multiple timepoints after surgery and processed for flow cytometry (Figure 1B, Supplemental Figure S1). During lesion development, the number of both CD4^+^ and CD8^+^ T-cells increased progressively (Figures 1C and 1D). In total, the absolute number of CD8^+^ T-cells increased 22-fold (*P <* 0.0001) at t = 28d compared with T-cells from native caval veins used as controls, while the absolute number of CD4^+^ T-cells increased 3-fold *(P* < 0.001). Specifically, at t = 28d, the absolute number of CD8^+^ effector-memory T-cells (Tem) (CD44^+^ CD62L^−^) increased 6-fold *(P =* 0.013) (Figure 1F) compared with baseline, while also the number of CD8^+^ central-memory T-cells (Tcm) (CD44^+^ CD62L^+^) increased (*P <* 0.001) (Figure 1H). In contrast, the absolute number of CD4^+^ Tem was not significantly increased (2-fold; *P =* 0.34) (Figure 1E), whereas the number of CD4^+^ Tcm did significantly increase at t = 28d compared with baseline (7-fold; *P =* 0.030) (Figure 1G). Altogether, these data demonstrate that primarily CD8^+^ T-cells accumulate in the atherosclerotic vein graft lesion during plaque development and to a lesser extent CD4^+^ T-cells. Additionally, CD8^+^ Tem and Tcm cells accumulate in greater number than CD4^+^ Tem and Tcm cells, indicating a more pronounced role for CD8^+^ T-cells in the development of vein graft atherosclerosis. Moreover, the T-cell dynamics in the circulation are dissimilar to the vein graft (Supplemental Figure S2), pointing to a localized effect.

### Expression of CD137 as well as CD69 by mainly CD8^+^ T-cells indicates T-cell activation during early stages of vein graft remodeling

**Figure 2:**
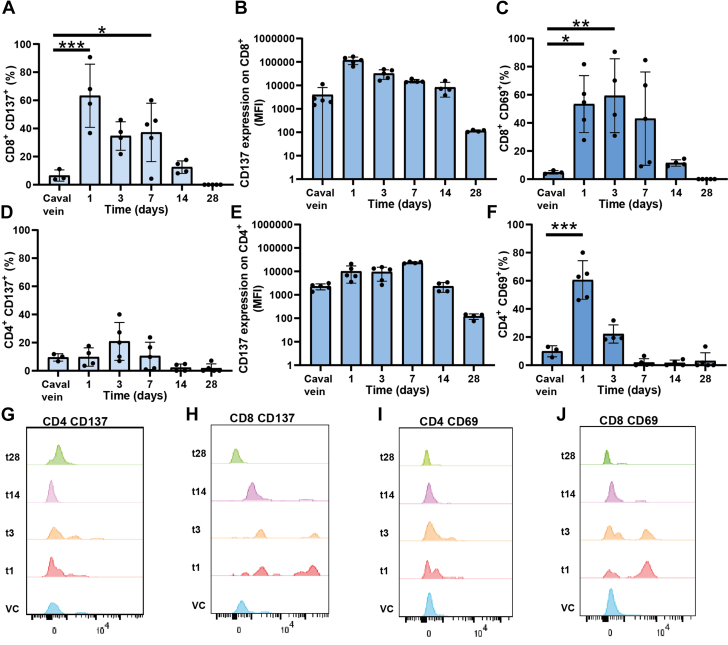
Expression of CD137 as Well as CD69 by Mainly CD8^+^ T-Cells Indicates T-Cell Activation During Early Stages of Plaque Development Vein graft, caval vein, spleen, lymph nodes, and blood samples were processed for flow cytometric analysis (n = 4-5 per group). Quantification of vein graft CD8^+^ T-cells expressing CD137 (% in A, mean fluorescent intensity [MFI] in B) and CD69 (C) as well as CD4^+^ T-cells expressing CD137 (% in D, MFI in E) and CD69 (F) (T-cells expressed as parent percentage). Representative histograms of CD137 and CD69 expression on CD4^+^ and CD8^+^ T-cells (G-J). Data is represented as mean ± SD and tested using 1-way analysis of variance with Dunnett post hoc test (A-F); significance is indicated for relevant comparisons and ∗*P <* 0.05, ∗∗*P <* 0.01, and ∗∗∗*P <* 0.001.

Having confirmed the accumulation of CD8^+^ and to a lesser extent CD4^+^ T-cells in atherosclerotic vein graft lesions over time, we assessed whether these lesional T-cells are activated, by analyzing cell-surface expression of the early activation marker CD69 and the costimulatory marker CD137, which is expressed within 24 hours after stimulation. Moreover, CD137 is expressed on mainly T-cells, but also endothelial cells in human atherosclerotic lesions,^33^^,^^34^ while we observe expression of CD137 by predominantly T-cells in human vein grafts (Supplemental Figure S3). Both CD69 and CD137 were expressed rapidly after surgery during early stages of lesion development (t = 1d, t = 3d, and t = 7d) (Figures 2A to 2F). The percentage of CD137^+^ cells increased significantly for CD8^+^ T-cells from baseline to t = 1d (*P <* 0.001), while the percentage CD137 expressing CD4^+^ T-cells did not increase significantly. After t = 1d, expression of CD137 gradually decreased over time until CD137 was hardly expressed on CD8^+^ T-cells at t = 28d. Additionally, the expression of CD137 (determined by mean fluorescent intensity [MFI]) was markedly higher on CD8^+^ T-cells compared with CD4^+^ T-cells (118861 vs 9450 on t = 1d, respectively) (Figures 2B, 2E, 2G, and 2H). Moreover, the expression of CD137 on CD8^+^ T-cells is far greater compared with what has previously been reported for other cell types (endothelial cells, macrophages, and vascular smooth muscle cells) (>20-fold, based on MFI).^25^ Together with colocalization of CD137 with predominantly CD3 in human vein graft tissue (Supplemental Figure S3), this suggests that CD137 affects mainly T-cells in vein graft remodeling. Furthermore, the significantly increased percentage and higher expression of CD137 on CD8^+^ T-cells compared with CD4^+^ T-cells indicates a more prominent role for CD8^+^ T-cells during vein graft remodeling in mice.

Expression of CD69 increased significantly from baseline to t = 1d for CD8^+^ T-cells *(P =* 0.014) and CD4^+^ T-cells (*P <* 0.001). The expression of CD69 peaked at t = 1d, when 53% of CD8^+^ T-cells and 61% of CD4^+^ T-cells expressed CD69. Expression of CD69 on CD4^+^ T-cells decreased rapidly thereafter, until virtual no expression at t = 7d, whereas CD8^+^ T-cells still expressed CD69 at t = 7d (Figures 2C, 2F, 2I, and 2J). Taken together, this suggests that both CD8^+^ T-cells and CD4^+^ T-cells are activated during early stages of vein graft remodeling. Expression of CD137 on CD8^+^ and CD4^+^ T-cells in other organs (blood, spleen, draining and nondraining—ie, inguinal—lymph nodes) was low compared with the CD137 expression on lesional T-cells (Supplemental Figures S4A and S4B). A significant increase in CD137^+^ CD8^+^ T-cells at t = 7d *(P =* 0.040) and t = 14d *(P =* 0.043) as well as CD137^+^ CD4^+^ T-cells at t = 14d (*P <* 0.001) and t = 28d (*P <* 0.001) in draining compared with nondraining lymph nodes was observed, indicating primarily local rather than systemic T-cell activation. Together with the profound expression of CD137 on CD8^+^ T-cells in vein grafts, this therefore provides a window for therapeutic targeting of lesional T-cells.

### Agonistic CD137 treatment increases lumen area and reduces lesion growth in atherosclerotic vein grafts

**Figure 3:**
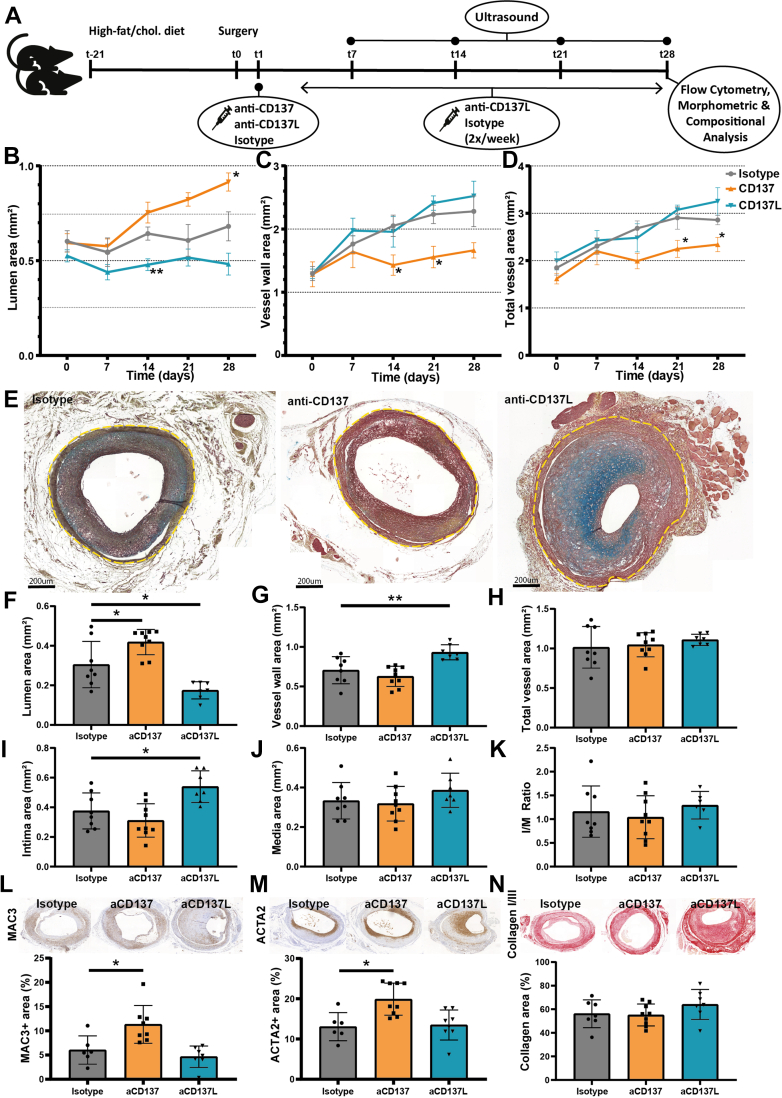
In vivo targeting of CD137: Agonistic Treatment Improves, Whereas Antagonistic Treatment Negatively Affects Vascular Remodeling Experimental set-up: high-fat/cholesterol-fed ApoE3∗Leiden mice were injected either isotype (n = 8), agonistic CD137 antibodies (aCD137, agonistic treatment) (n = 9) or CD137L blocking antibodies (aCD137L, antagonistic treatment) (n = 7). Treatment was started at t = 1d: aCD137 was administered once, while isotype and aCD137L were administered twice per week until sacrifice (A). Quantification of lumen area (B), vessel wall area (C), and total vessel area (D) of atherosclerotic vein grafts using ultra-high frequency ultrasound. Representative images of Movat Pentachrome-stained atherosclerotic vein graft lesions from mice treated with isotype, aCD137, and aCD137L (E). Quantification of histology: lumen (F), vessel wall (G), total vessel (lumen + lesion) (H), intima (I), and media area (J) in mm^2^, Intima/media ratio (K). Representative images and quantification of MAC3 (L), ACTA2 (M), and collagen staining (N) (normalized to total vein graft area). Data are presented as mean ± SEM and were tested using 2-way repeated measures analysis of variance or mixed-effects analysis (B-D) with repeated measures and Dunnett post hoc test. Data are presented as mean ± SD and were tested using 1-way analysis of variance or Kruskal-Wallis with Dunnett or Dunn post hoc test, respectively (F-N). ∗*P <* 0.05, ∗∗*P <* 0.01, and ∗∗∗*P <* 0.001.

To examine how immunomodulation by agonistic or antagonistic CD137 treatment affects vein graft atherosclerosis, mice were treated with either agonistic CD137 antibodies that induce T-cell activation (agonistic treatment, aCD137) or CD137L blocking antibodies to reduce T-cell activation (antagonistic treatment, aCD137L) (Figure 3A). Mice were randomized based on age and plasma cholesterol levels (Supplemental Figure S5). Both agonistic and antagonistic CD137 targeting was well tolerated and no significant differences in body weight were observed between all groups (Supplemental Figure S4).

Ultra-high frequency ultrasound imaging was used to longitudinally quantify vascular remodeling (Figure 3B). Morphometric area measurements were performed on 2-dimensional images generated from 3-dimensional volume reconstructions (Supplemental Figure S6). At t = 0 (immediately postoperatively), no significant differences in lumen, vessel wall, or total vessel area (lumen + vessel wall) were observed. At t = 28d, lumen area was significantly increased by 34% upon agonistic CD137 treatment compared with isotype-control *(P =* 0.046) (Figure 3B). Furthermore, agonistic CD137 treatment reduced vessel wall area by 30% at t = 14d, t = 21d, and t = 28d *(P =* 0.033; *P =* 0.021; and *P =* 0.079) (Figure 3C). Overall, this led to a significant 23% and 18% decrease in total vessel area compared with isotype-control at t = 21d and t = 28d *(P =* 0.018 and *P =* 0.022) (Figure 3D). Contrary to agonistic treatment, antagonistic CD137 treatment reduced lumen area by 25% at t = 14d *(P =* 0.006) and 29% at t = 28d *(P =* 0.104) (Figure 3C), whereas no differences in wall area or total vessel area where observed compared with control. Together these data indicate a reduction in vein graft atherosclerosis in response to agonistic CD137 treatment, while blocking CD137 reduced lumen area.

Histology at t = 28d (Figure 3E) confirmed ultrasound morphometric analysis, demonstrating that lumen area was significantly increased by 37% *(P =* 0.018) upon agonistic CD137 treatment compared with control, whereas antagonistic CD137 treatment resulted in a significant decrease of 43% in lumen area compared with isotype-control *(P =* 0.011) (Figure 3F). The vessel wall area of mice receiving antagonistic CD137 treatment was increased significantly by 32% compared with isotype control *(P =* 0.008) (Figure 3G). Total vessel area was not different between different treatment groups (Figure 3H). The increase in vessel wall area upon antagonistic CD137 treatment was derived from a 44% significant increase in the intimal area *(P =* 0.021) (Figure 3I), while neither media area (Figure 3J) nor the intima/media ratio (Figure 3K) differed compared with isotype-control. Next, we assessed whether the composition of the atherosclerotic vein graft lesions was affected by treatment. MAC3^+^ area was 88% increased *(P =* 0.011), whereas ACTA2^+^ area was more than doubled upon agonistic CD137 treatment *(P =* 0.008), indicating a relative increase in macrophages and vascular smooth muscle cells (VSMCs) (Figures 3L and 3M). The relative area covered by collagen, the most abundant extracellular matrix component produced by VSMCs in vein graft atherosclerosis, was comparable between all treatment groups (Figure 3N). Altogether, the increase in lumen area and decrease in lesion size indicate improved vascular remodeling upon agonistic CD137 treatment and aggravated vascular remodeling in response to antagonistic CD137 treatment.

### Agonistic CD137 treatment induces long-lasting effector-memory CD8^+^ T-cell formation in the blood circulation and increases systemic IFN-γ production

**Figure 4:**
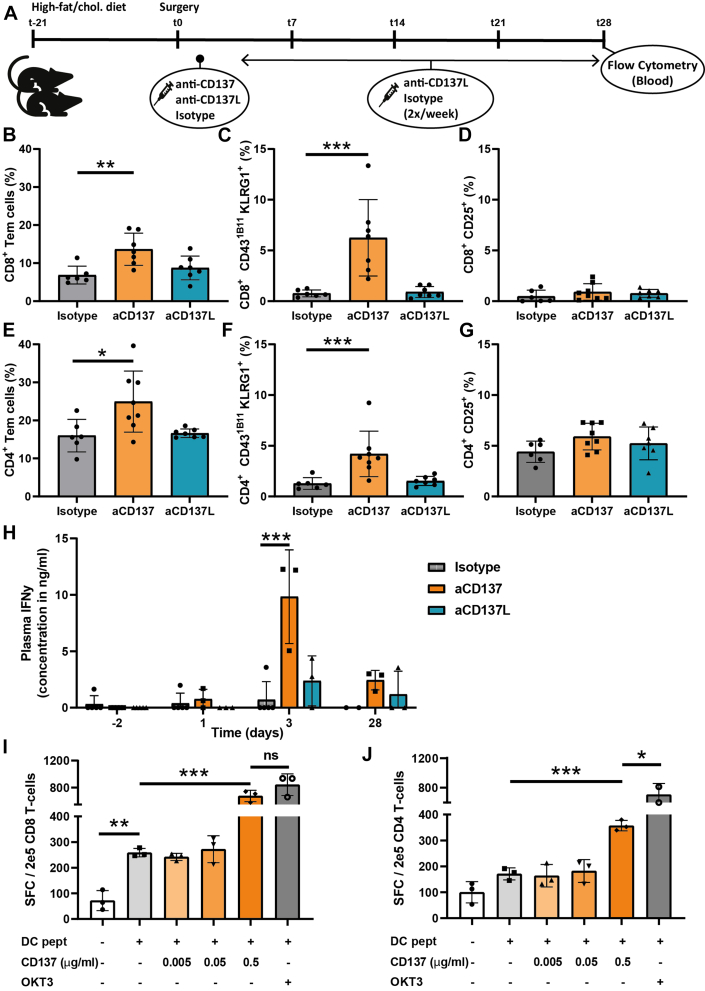
Agonistic CD137 Treatment Induces Activation of Primarily Circulating CD8^+^ T-Cells and Augments IFN-γ Production In Vivo as Well as In Vitro Flow cytometric analysis on blood of separate mice harvested at t = 28d (A), assessing CD8^+^ T-cell differentiation (B), (co-)expression of CD43^1B11^ KLRG1 (C), as well as CD25 (D) on the parental CD8^+^ T-cells. Additionally, CD4^+^ T-cell differentiation (E) and (co-)expression of CD43^1B11^ KLRG1 (F) as well as CD25 (G) on the parental CD4^+^ T-cells were analyzed after isotype or (ant)agonistic CD137 treatment. Plasma IFN-γ measurements (H). Quantification of IFN-γ ELISpot assay: number of spot-forming colonies (SFC) of human CD8^+^ (I) and CD4^+^ T (J) cells cultured in the presence of autologous peptide-pulsed dendritic cells (DCs), agonistic CD137 antibodies and OKT3. For this in vitro experiment, the means of 3 individual experiments consisting of 3 different donors are shown. Data is represented as mean ± SD and tested using 1-way analysis of variance or Kruskal-Wallis with Dunnett or Dunn post hoc test, respectively (B-G), 1-way analysis of variance with Sidak’s post hoc test (I and J), and 2-way analysis of variance with repeated measures and Dunnett post hoc test (H) and ∗*P <* 0.05, ∗∗*P <* 0.01, and ∗∗∗*P <* 0.001.

Next, the effect of agonistic CD137 and antagonistic CD137L treatment on circulating T-cells was evaluated at the t = 28d timepoint by flow cytometric analysis (Figure 4A, Supplemental Figure S7). The percentage of circulating CD4^+^ (25% vs 16%; *P =* 0.14) as well as CD8^+^ (14% vs 7%; *P =* 0.004) Tem cells were significantly increased after agonistic CD137 treatment (Figures 4B and 4E).

The activation status of circulating effector-memory T-cells was further assessed by the effector T-cell markers KLRG1 and CD43^1B11^. The 1B11 antibody clone (CD43^1B11^) recognizes the O-glycosylated form of CD43, which is only expressed by (activated) effector T-cells.^35^ Agonistic CD137 treatment induced T-cell activation, as demonstrated by a significant 8-fold increase of CD8^+^ T-cells expressing KLRG1 as well as CD43^1B11^ (*P <* 0.001) (Figure 4C). In addition, KLRG1^+^ CD43^1B11^ CD4^+^ T-cells were increased by 3.3-fold upon agonistic CD137 treatment *(P =* 0.002) (Figure 4F). In contrast, there were no differences in expression of CD25 on both CD4^+^ and CD8^+^ T-cells, indicating that at later timepoints no recent activation occurs (Figures 4D and 4G). In addition to the increased number of activated circulating T-cells, agonistic CD137 treatment also resulted in a 13-fold increase in plasma IFN-γ concentration compared with isotype-controls at t = d3 (*P <* 0.001) (Figure 4H).

To assess whether agonistic CD137 treatment affects IFN-γ production by human CD4^+^ or CD8^+^ T-cells, these cells were cultured in vitro with autologous peptide-pulsed DCs. Human CD8^+^ T-cells treated with 0.5 μg/mL, but not 0.005 and 0.05 μg/mL, agonistic CD137 antibody exhibited a 2.6-fold increase in the number of spot-forming colonies (*P <* 0.001), in contrast to CD4^+^ T-cells that only exhibited a 1.7-fold increase when treated with the same concentration agonistic CD137 antibody (*P <* 0.001) (Figures 4I and 4J). Together with the systemic increase of mainly activated CD8^+^ T-cells, this indicates that agonistic CD137 treatment primarily activates CD8^+^ T-cells and only to a lesser extent CD4^+^ T-cells, while also significantly augmenting IFN-γ production.

### Increased activation of lesional CD4^+^ and CD8^+^ T-cells upon agonistic CD137 treatment

**Figure 5:**
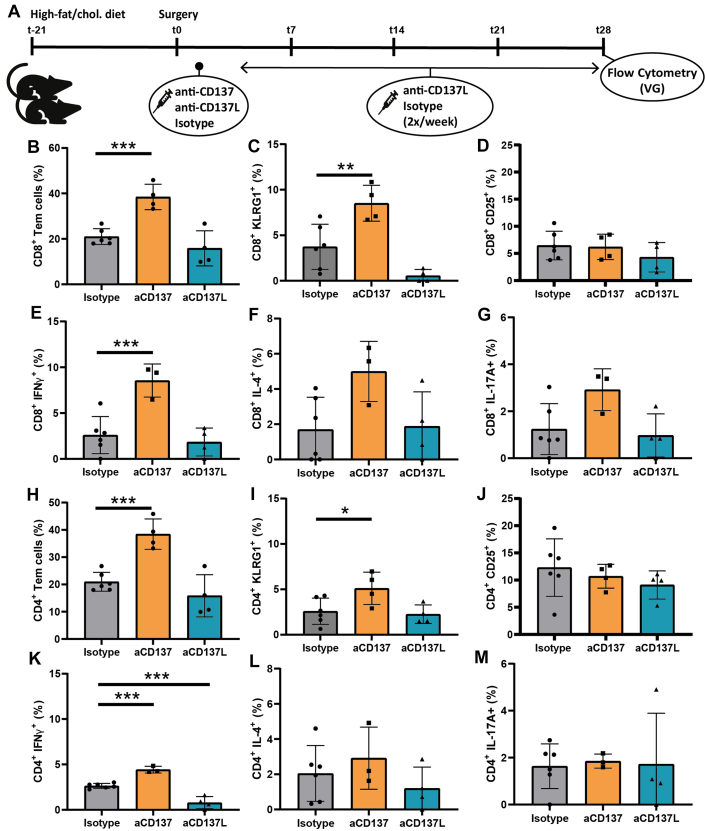
T-Cell Differentiation and Polarization in Atherosclerotic Vein Grafts After Treatment With Agonistic or Antagonistic CD137 Treatment Experimental set-up: high-fat/cholesterol-fed ApoE3∗Leiden mice were injected either isotype (n = 6), agonistic CD137 antibodies (aCD137, agonistic treatment) (n = 4) or CD137L blocking antibodies (aCD137L, antagonistic treatment) (n = 4), and at t = 28d, vein grafts were harvested and processed for flow cytometric analysis (A). Flow cytometric analysis assessing CD8^+^ T-cell differentiation (B), (co-)expression of CD43^1B11^ KLRG1 (C), CD25 (D), IFN-γ (E), IL-4 (F) and IL-17A (G) on CD8^+^ T-cells. Additionally, CD4^+^ T-cell differentiation (H) and (co-)expression of CD43^1B11^ KLRG1 (I), CD25 (J), IFN-γ (K), IL-4 (L) and IL-17A (M) on CD4^+^ T-cells were analyzed after isotype or (ant)agonistic CD137 treatment. Data is represented as mean ± SD and statistically tested by 1-way analysis of variance with Dunnett post hoc test, ∗*P <* 0.05, ∗∗*P <* 0.01, and ∗∗∗*P <* 0.001. VG = vein graft.

To examine how immune modulation by agonistic or antagonistic CD137 treatment affects CD4^+^ and CD8^+^ T-cell polarization in the atherosclerotic lesion, mice (n = 4-6/group) underwent vein graft surgery and were subjected to the (similar) agonistic or antagonistic CD137 treatment regimen after which the vein grafts were harvested and processed for flow cytometry at t = 28d (Figure 5A, Supplemental Figure S8). Agonistic treatment-induced differentiation of lesional CD8^+^ T-cells toward Tem cells (*P <* 0.001) (Figure 5B), which was accompanied by a 2-fold increase in CD8^+^ KLRG1^+^ T-cells *(P =* 0.006) (Figure 5C). In contrast, antagonistic CD137 treatment reduced the number CD8^+^ KLRG1^+^ T-cells *(P =* 0.058). Similar to the circulating T-cells, no differences in expression of CD25 were found on lesional CD8^+^ T-cells (Figure 5D), once more demonstrating that agonistic CD137 treatment induces temporal T-cell activation, primarily during early stages of plaque development.

In addition, agonistic CD137 treatment also affected T-cell polarization. A significant 3-fold increase in lesional CD8^+^ IFN-γ^+^ T-cells *(P =* 0.002) compared with isotype-control was observed upon agonistic CD137 treatment (Figure 5E). Furthermore, the amount of CD8^+^ IL-4^+^ T-cells was also increased by almost 3-fold *(P =* 0.056) (Figure 5F), while the number CD8^+^ IL-17A^+^ T-cells was also more than doubled after agonistic CD137 treatment *(P =* 0.071) (Figure 5G). In contrast, antagonistic CD137 treatment did not affect expression of IFN-γ, IL-4, or IL-17A by lesional CD8^+^ T-cells. Similar to CD8^+^ T-cells, agonistic CD137 treatment resulted in increased number of CD4^+^ Tem cells compared with isotype-control *(P =* 0.007) (Figure 5H). Furthermore, a concomitant 2-fold increase in KLRG1^+^ CD4^+^ T-cells was observed *(P =* 0.038) (Figure 5I), indicating that agonistic CD137 treatment did not only induce CD8 but also CD4 Tem formation, albeit to a lesser extent. Antagonistic CD137 treatment did not affect the percentage of CD4^+^ T-cells expressing KLRG1 in the plaque. Following a pattern similar to CD8^+^ T-cells, no differences in CD25 expression were observed on CD4^+^ T-cells (Figure 5J) for both agonistic as well as antagonistic CD137 treatment. The effects of treatment on CD4^+^ T-cells are maintained until later stages of plaque development, as demonstrated by increased expression of IFN-γ (*P <* 0.001), but not IL4 and IL-17A in CD4^+^ T-cells upon agonistic CD137 treatment at t = 28d. In contrast, antagonistic CD137 treatment reduced IFN-γ (*P <* 0.001), but not IL-4 and IL-17A expression by CD4^+^ T-cells compared with isotype-control (Figures 5K to 5M). Moreover, within the same mice these effects were not observed in other lymphoid organs (Supplemental Figures S9 and S10) or in circulating T-cells (Supplemental Figure S11). All in all, agonistic CD137 treatment activates T-cells during early stages of plaque development, whilst also inducing KLRG1 expression on CD8^+^ effector T-cells. Taken together, these results demonstrate that agonistic CD137 treatment primarily affects CD8^+^ T-cells, and only to a lesser extent CD4^+^ T-cells. Additionally, although we were able to detect systemic effects of CD137 treatment (Figure 4), which might affect the observed effects in the vein graft, the discrepancy between paired vein graft (Figure 5) and blood samples (Supplemental Figure S11) is suggestive of a pronounced local effect.

### Agonistic CD137 treatment suppresses intraplaque angiogenesis, while stimulating neovessel maturation and increasing CXCL10

**Figure 6:**
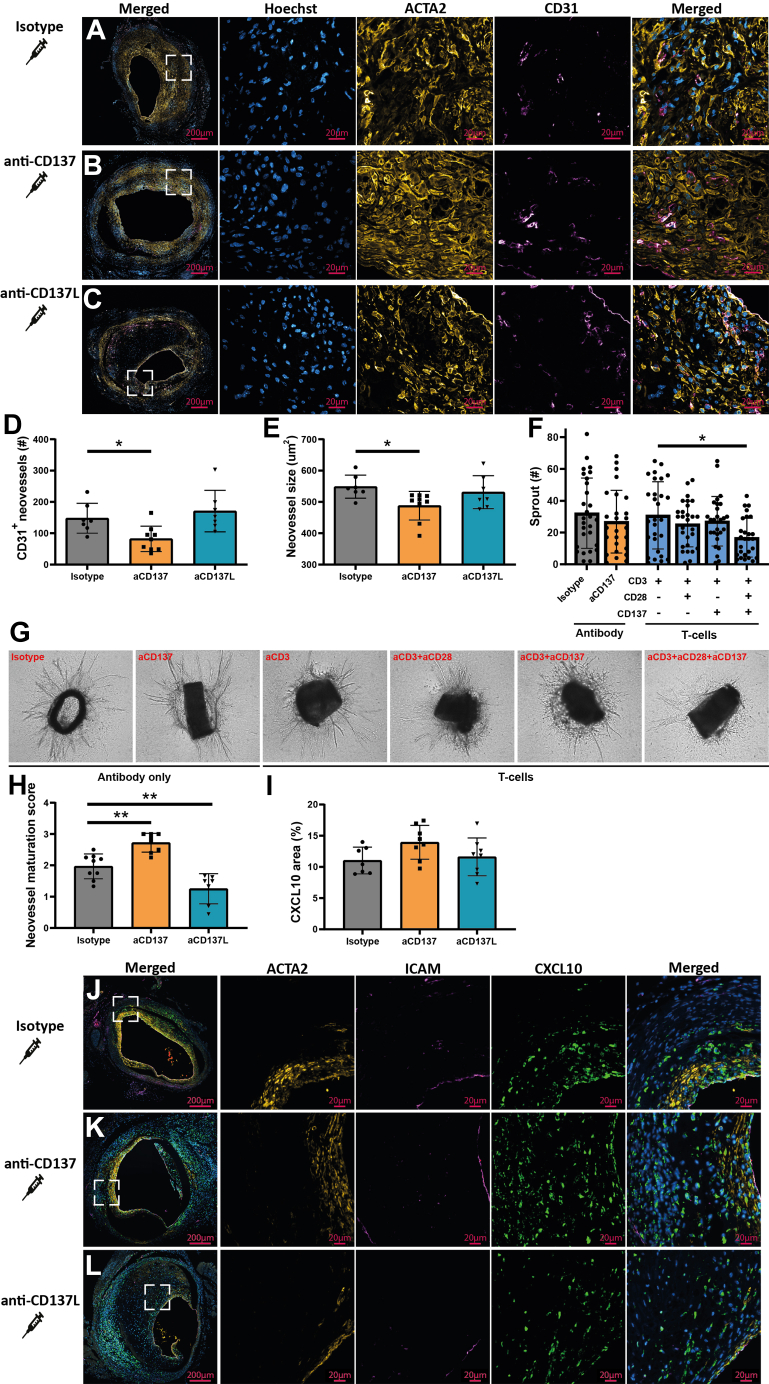
Intraplaque Angiogenesis Is Inhibited Upon Agonistic CD137 Treatment, While Neovessel Maturation Is Improved Representative images of ACTA2, CD31, and Hoechst staining of atherosclerotic vein graft lesions from mice receiving isotype (n = 7) (A), agonistic CD137 (n = 8) (B), or antagonistic CD137 treatment (n = 7) (C). Quantification of absolute number of neovessels (D) and neovessel size (E). Representative images (F) and quantification of aortic ring assay (G). Maturation of the neovessels, as assessed by neovessel coverage by ACTA2^+^ cells (H). Representative images of CXCL10 and Hoechst stain of atherosclerotic vein graft lesions from mice receiving isotype (n = 7) (J), agonistic CD137 (n = 9) (K), or antagonistic CD137 treatment (n = 7) (L). Quantification of CXCL10^+^ vein graft area (I). Data is represented as mean ± SD and tested using 1-way analysis of variance with Dunnett post hoc test (D, E, H and I), 1-way analysis of variance with Sidak’s post hoc test (F) and ∗*P <* 0.05, ∗∗*P <* 0.01, and ∗∗∗*P <* 0.001.

Neovessels growing into the hypoxic atherosclerotic plaque are a hallmark of unstable lesions.^36^^,^^37^ These neovessels are immature and therefore commonly associated with plaque progression. Additionally, inflammation is a trigger for intraplaque angiogenesis. We have previously identified CD3^+^ T-cells in close proximity to these neovessels,^26^ and in our current study, also observe both CD4^+^ and CD8^+^ T-cells localizing near lesional neovessels (Figure 1A). Therefore, we examined the effect of CD137 treatment on intraplaque angiogenesis (Figures 6A to 6C). Agonistic CD137 treatment significantly reduced the neovessel density by 45% compared with isotype-control *(P =* 0.036) (Figure 6D). Furthermore, the average neovessel size decreased by 11% significantly compared with isotype-control upon agonistic CD137 treatment *(P =* 0.030) (Figure 6E). In sum, this indicates that agonistic CD137 treatment suppresses intraplaque angiogenesis. In contrast, antagonistic CD137 treatment did not affect neovessel density or size (Figures 6D and 6E).

To assess whether the reduction in neovascularization originates from a direct or indirect (ie, through CD8^+^ T-cells) effect of CD137, we performed an aortic ring experiment.^38^ Direct stimulation of aortic rings with anti-CD137 did not affect the number of sprouts that were growing from the aortic ring compared with isotype (Figures 6F and 6G). Next, we cocultured the aortic rings with murine CD8^+^ T-cells. Before the coculture, the T-cells were incubated with(out) anti-CD137 as well as anti-CD3 and/or anti-CD28, because sole stimulation with these individual antibodies does not result in T-cell activation in vitro to a similar extent as in inflammatory in vivo conditions.^39^ Interestingly, the number of sprouts was significantly reduced when T-cells were prestimulated with anti-CD3, anti-CD28, and anti-CD137. This indicates that CD137-activated CD8^+^ T-cells can inhibit angiogenesis, whereas anti-CD137 treatment without T-cells does not affect angiogenesis.

In addition to the quantity of neovessels, the quality of these neovessels, such as pericyte coverage, is important. Activated CD8^+^ IFN-γ^+^ and CD4^+^ IFN-γ^+^ T-cells can induce the proliferation of VSMC-like cells such as pericytes, which are essential for the maturation of neovessels.^40^ Coverage of plaque neovessels with ACTA2 was significantly increased upon agonistic *(P =* 0.002), but significantly decreased upon antagonistic CD137 treatment compared with isotype-control *(P =* 0.003) (Figure 6H). This suggests that agonistic CD137 not only inhibits the number of neovessels, but also improves the quality rendering a more stable plaque phenotype.

To delineate a potential mechanism underlying the decrease in intraplaque angiogenesis after agonistic CD137 treatment, vein grafts were costained for CXCL10, ACTA2, and ICAM (Figures 6I to 6L), because it is known that CXCL10 inhibits angiogenesis and is up-regulated in response to IFN-γ.^41^ Lesional CXCL10^+^ area was nonsignificantly increased by 26% upon agonistic CD137 treatment compared with control *(P =* 0.085) (Figure 6K). No differences were observed upon antagonistic CD137 treatment.

## Discussion

In this study, we assessed the T-cell immune landscape of accelerated atherosclerotic vein graft lesions and observed a progressive increase in the absolute number of CD4^+^ and CD8^+^ T-cells, specifically effector memory T-cells, as the atherosclerotic lesion develops. CD8^+^ T-cells and also CD4^+^ T-cells, albeit to a lesser extent, are activated during early stages of vein graft atherosclerosis. We identified CD137 as a key costimulatory molecule that is expressed on plaque CD8^+^ T-cells during this period, and targeting of the CD137-CD137L signaling pathway by administering agonistic antibodies induced lesional T-cell differentiation toward effector-memory, while significantly increasing lumen area of the vein graft. In contrast, antagonistic CD137 treatment decreased lumen area and promoted lesion growth. Strikingly, agonistic CD137 targeting impeded intraplaque angiogenesis, while improving neovessel maturation, whereas antagonistic CD137 treatment decreased neovessel maturation. Furthermore, direct agonism of CD137 did not inhibit angiogenesis, while (CD137-) activated CD8^+^ T-cells did reduce neovascularization.

The progressive increase in the absolute number of T-cells into the atherosclerotic vein graft during plaque development is preceded by rapid expression of CD69 (CD4^+^ and CD8^+^ T-cells) as well as expression of CD137 (CD8^+^ T-cells), which are only expressed during the initial stages of vein graft remodeling. The expression of CD69 and CD137 indicates T-cell activation rapidly after surgery during early stages of vein graft remodeling. This is also corroborated by the progressive increase in the number of Tem and Tcm over time in the vessel wall. Although CD137 is also expressed on other cell types in the atherosclerotic plaque, we found that the expression of CD137 on CD8^+^ T-cells is far greater compared with what has previously been reported for endothelial cells, macrophages, and VSMC (>20-fold, based on MFI), while CD137 colocalized mainly with CD3^+^ cells in human vein grafts.^25^ Additionally, direct agonism of CD137 did not affect angiogenesis in vitro, while CD137-activated CD8^+^ T-cells did significantly inhibit neovascularization. Overall, these data suggest that the effects of CD137 treatment are mainly exerted through T-cells, and only to a lesser extent through other cell types that express CD137. Furthermore, administration of agonistic CD137 antibodies at t = 1d, also augmented the differentiation of both circulating as well as lesional T-cells into Tem, which was observed at t = 28d. Interestingly, the discordance between lesional and blood T-cells suggests a local effect in the vein graft on top of the systemic effects.

Additionally, agonistic CD137 treatment increased expression of IFN-γ on plaque T-cells, while systemic IFN-γ concentration was also increased shortly after agonistic CD137 treatment. This was corroborated by a strong increase in IFN-γ production in vitro for both CD4^+^ and CD8^+^ T-cells upon stimulation with CD137. Comparable responses, inducing T-cell proliferation, memory formation, and activation, have been demonstrated by monoclonal agonistic CD137 antibodies to eradicate established tumors.^42^, ^43^, ^44^, ^45^ Moreover, agonistic CD137 treatment has been shown to preferentially target CD8^+^ T-cells, which we also observe, and markedly enhances IFN-γ production.^46^ In murine models for spontaneous atherosclerosis, stimulation of CD137 promoted increased IFN-γ production and expression in atherosclerotic lesions.^34^^,^^47^ Similarly, genetic (nonspecific) silencing of CD137 decreased IFN-γ expression on T-cells in hyperlipidemic mice.^48^ Furthermore, agonistic CD137 treatment accelerates atherogenesis,^34^ whereas genetic silencing reduces atherosclerotic plaque size.^48^ This appears contradictory to our findings that agonistic CD137 treatment reduces vein graft atherosclerosis, while antagonistic CD137 treatment increased lesion size in our vein grafts. Spontaneous atherosclerosis is characterized by chronic, low-grade inflammation.^49^ In contrast, vein graft atherosclerosis is characterized by an additional period of highly acute, high-grade inflammation caused by surgical injury and perturbed hemodynamics.^50^, ^51^, ^52^, ^53^ Moreover, the expression of CD137 and CD69 at t = 1d suggests surgery-related activation of T-cells, because the earliest signs of foam cell/atherosclerotic plaque formation are observed at t = 7d.^54^ To a certain extent, this inflammatory response is required to facilitate adaptation of the vein graft to the arterial blood flow.^55^ The inflammation, however, rapidly diminishes after surgery and only reappears at later stages of vein graft remodeling in a similar chronic, low-grade inflammation that is similar to what is observed in spontaneous atherosclerosis.^56^ Administering agonistic CD137 antibodies at t = 1d—when CD137 expression peaked—resulted in immunomodulation during this initial phase of inflammation after surgery to induce outward (beneficial) remodeling. These findings further strengthen earlier observations that the early postoperative period is critical for vein graft remodeling, because 28 days immunomodulation by dexamethasone did not provide additional protection from adverse vein graft remodeling compared with 7 days postsurgical immunomodulation.^57^

The effects of early immunomodulation by (ant)agonistic on vein graft morphometry became apparent from t = 14d onwards. Agonistic CD137 treatment increased lumen size while concomitantly preventing excessive vessel wall thickening, whereas antagonistic CD137 treatment decreased lumen size. This effect coincides with the onset of the chronic, low-grade inflammation in the vessel wall as well as the ingrowth of neovessels from the vasa vasorum into the developing atherosclerotic plaque, which occurs from approximately t = 10d.^26^^,^^58^ These intraplaque neovessels often lack pericyte coverage and are strongly associated with atherosclerotic plaque instability.^59^ In our current study, we found that agonistic CD137 treatment decreased the number of neovessels, while increasing pericyte coverage, which indicates improved plaque stability. In vitro, we observed that agonistic CD137 antibodies did not diminish neovascularization directly, but only through CD8^+^ T-cells, indicating that T-cells are critical in regulating intraplaque angiogenesis. T-cells can control angiogenesis through cytokine and chemokine secretion.^60^ Stimulation of CD137 signaling profoundly increases IFN-γ expression on T-cells,^46^ which we also observed on lesional T-cells at t = 28d. This suggests that the immunomodulation by CD137 antibodies during the initial, high-grade inflammatory phase after surgery, also affected the chronic, low-grade inflammation at later stages of vein graft remodeling.

IFN-γ has been described to directly inhibit proliferation and migration of human endothelial cells.^61^^,^^62^ Furthermore, IFN-γ down-regulates VEGF, but up-regulates, amongst others, CXCL10.^63^^,^^64^ This chemokine inhibits angiogenesis and has been described to stimulate neovessel maturation by enhancing pericyte recruitment to neovessels.^65^ Agonistic CD137 treatment increases CXCL10 production by T-cells, but not by myeloid cells.^66^ Moreover, activation of CD4^+^ IFN-y^+^ T-cells has been shown to improve neovessel maturation.^67^ Here, we observed a trend toward increased CXCL10 protein expression in the lesion upon agonistic CD137 treatment, in addition to the increase in lesional CD4^+^ and CD8^+^ IFN-γ^+^ T-cells. Whether there is a causal relation between the increased presence of plaque IFN-γ^+^ T-cells and reduced intraplaque neovascularization remains to be investigated. Overall, however, agonistic CD137 treatment resulted in reduced intraplaque angiogenesis, while concomitantly improving neovessel maturation, yielding improved lesion stability, whereas antagonistic CD137 treatment decreased neovessel maturation.

Altogether, we demonstrate an important role of T-cells in vein graft atherosclerosis and identify CD137-CD137L signaling as a potential therapeutic target for immunomodulation to improve vein graft remodeling. Agonistic CD137 treatment increased lumen area, whereas antagonistic CD137 treatment decreased lumen area and aggravated atherosclerotic plaque development. Mechanistically, agonistic CD137 treatment induced T-cell differentiation into long-lasting effector-memory subsets and increased production of IFN-γ. Furthermore, agonistic CD137 improved plaque neovascularization by decreasing the number of neovessels while increasing their maturation. Altogether, this illustrates that CD137 costimulation plays a critical role in vein graft atherosclerosis. The up-regulation of CD137 on T-cells located in the vessel wall during early stages of plaque development, renders this molecule a potential target for early immunomodulation to improve vein graft remodeling and reduce atherosclerotic plaque development by inhibiting intraplaque angiogenesis.

Perspectives **COMPETENCY IN MEDICAL KNOWLEDGE:** Various T-cell subsets accumulate over time in murine, unstable atherosclerotic vein graft lesions, while their activation occurs rapidly after surgery. CD137-CD137L signaling regulates activation and differentiation on these T-cells, which in turn affect intraplaque angiogenesis and atherosclerotic lesion development.

**TRANSLATIONAL OUTLOOK 1:** Delineating the contribution of T-cells to the development of vein graft atherosclerosis and intraplaque angiogenesis is of importance to identify potential therapeutic targets to attenuate adverse vein graft remodeling.

**TRANSLATIONAL OUTLOOK 2:** Improved vein graft remodeling and diminished intraplaque angiogenesis upon agonistic CD137 treatment demonstrated in this study suggest CD137 as a promising target required to be investigated in larger animal models and clinical studies to reduce vein graft failure.

## Funding Support and Author Disclosures

This project was supported by a LUMC MD-PhD grant to Dr de Jong and a Rembrandt Institute for Cardiovascular Sciences as well as a Public Private Partnership grant ‘NIPAC’ to Dr de Vries. All other authors have reported that they have no relationships relevant to the contents of this paper to disclose.
