# Supplementary material for: CD137 Signaling Modulates Vein Graft Atherosclerosis by Driving T-Cell Activation and Regulating Intraplaque Angiogenesis
Source: JACC Basic Transl Sci. 2025 Jul 29;10(8):101323. doi: 10.1016/j.jacbts.2025.101323 (PMC12332866; doi:10.1016/j.jacbts.2025.101323)
Supplement: Supplemental Figures 1-11 [file mmc1.docx]

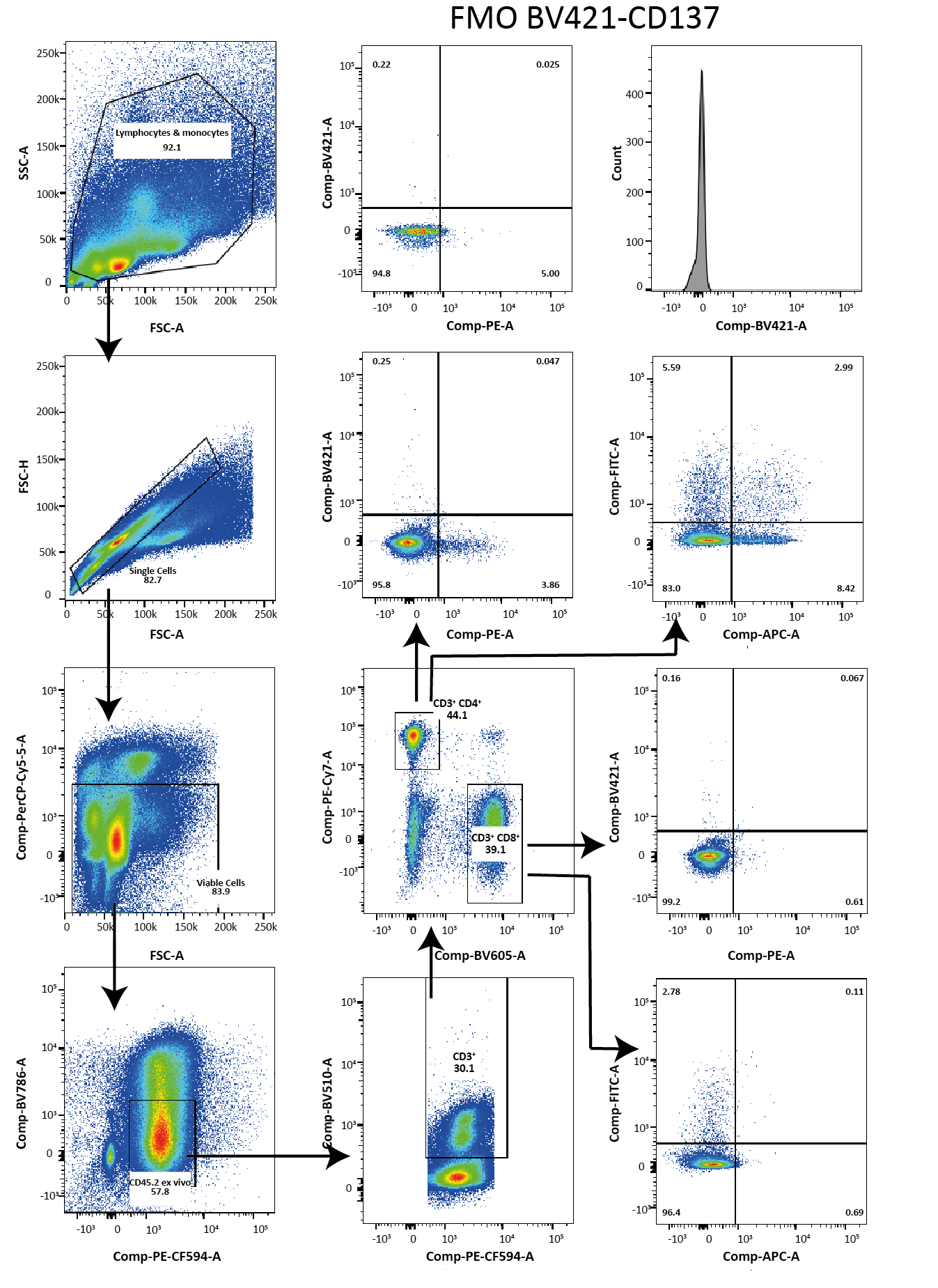
**Supplemental figures**


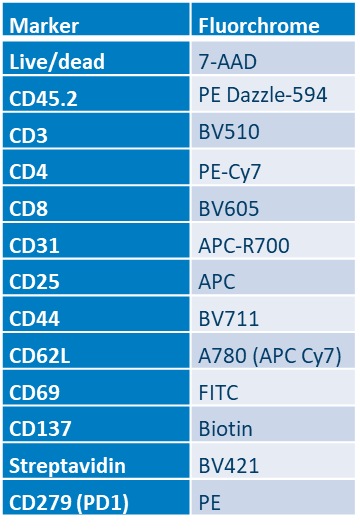


**Figure S1 | Flow cytometry gating strategy investigating the immune landscape of lesional CD8^+^ and CD4^+^ T cell from atherosclerotic vein graft lesions.** Representative example of gating strategy used to identify expression of CD69 and CD137 on lesional CD4^+^ and CD8^+^ cells. Single cell suspensions from atherosclerotic vein grafts (n=4-5/timepoint) were obtained by enzymatic digestion and consequently stained for flow cytometric analysis.

**
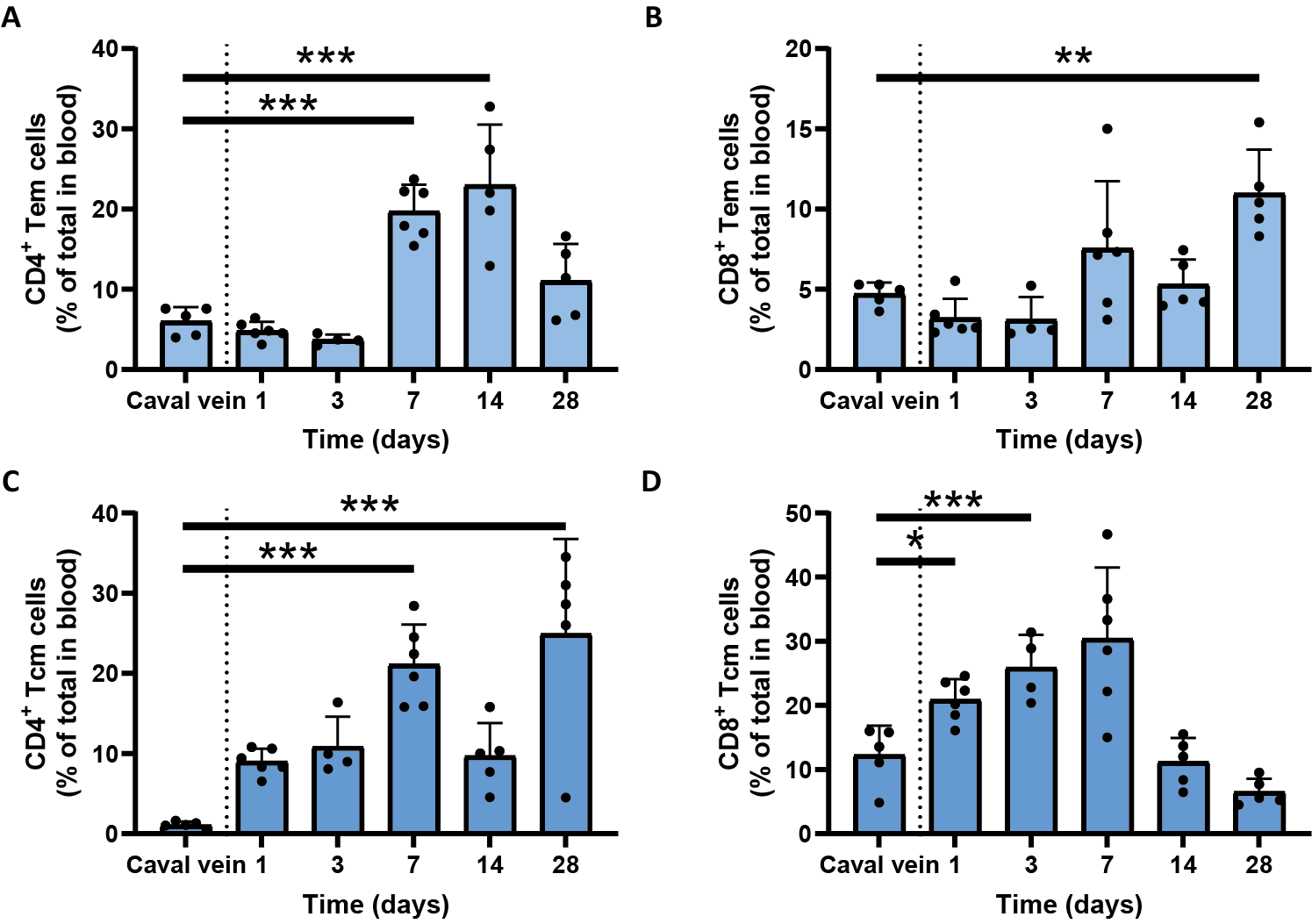
**

**Figure S2 | CD4 and CD8 T cell dynamics in blood at multiple timepoints after vein graft surgery.** Experimental setup: caval veins and vein grafts of high-fat/cholesterol-fed ApoE3*Leiden mice (n=3-4 per timepoint were harvested at different timepoints and processed for flow cytometry. Quantification of flow cytometric analysis: relative number of CD4^+^ and CD8^+^ effector-memory T cells (Tem, CD62L**^-^** CD44**^+^**) (**A**,**B**) and central memory T cells (Tcm, CD62L**^+^** CD44**^+^**) (**C**,**D**) in blood. Tem; effector-memory T cells (CD62L**^-^** CD44**^+^**), Tcm; central memory T cells (CD62L**^+^** CD44**^+^**). Data were represented as mean ± SD and tested using one-way ANOVA with Dunnett post-hoc test, significance is indicated for relevant comparisons, and *p<0.05, **p<0.01, and ***p<0.001.


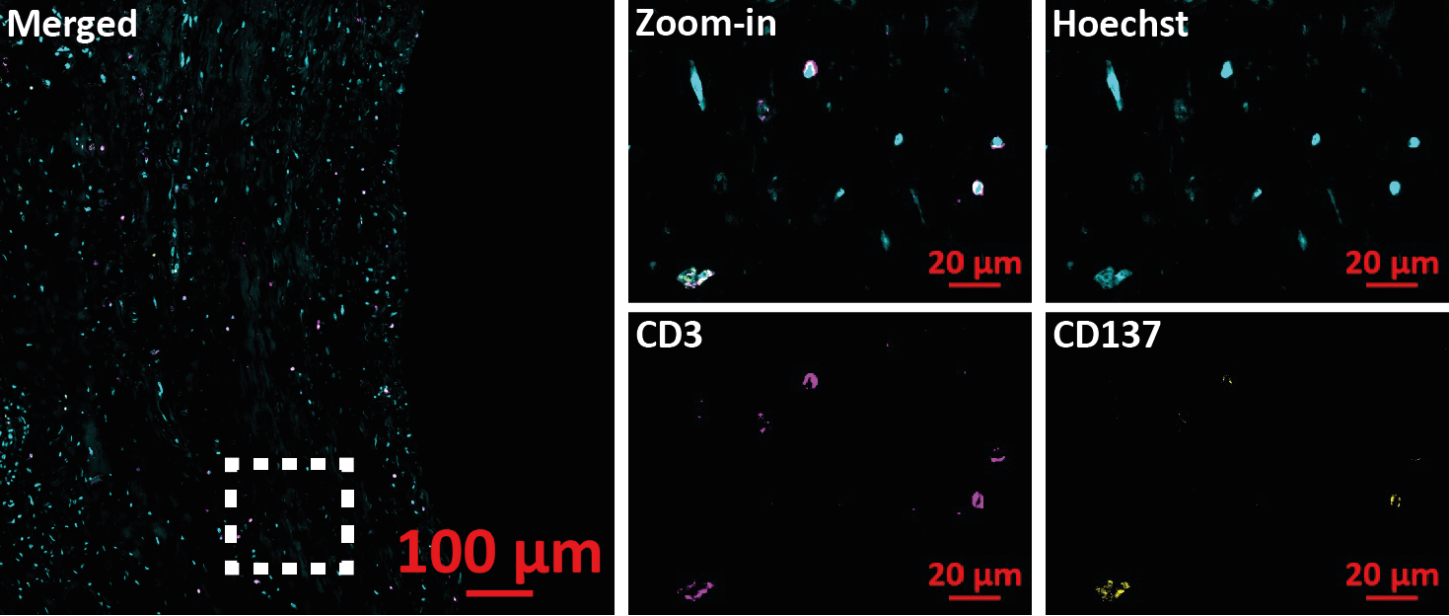


**Figure S3 | CD137 is mainly expressed on CD3^+^ cells in human vein grafts**

Representative images of CD3, CD137 and Hoechst staining of human vein graft lesions (n=4)


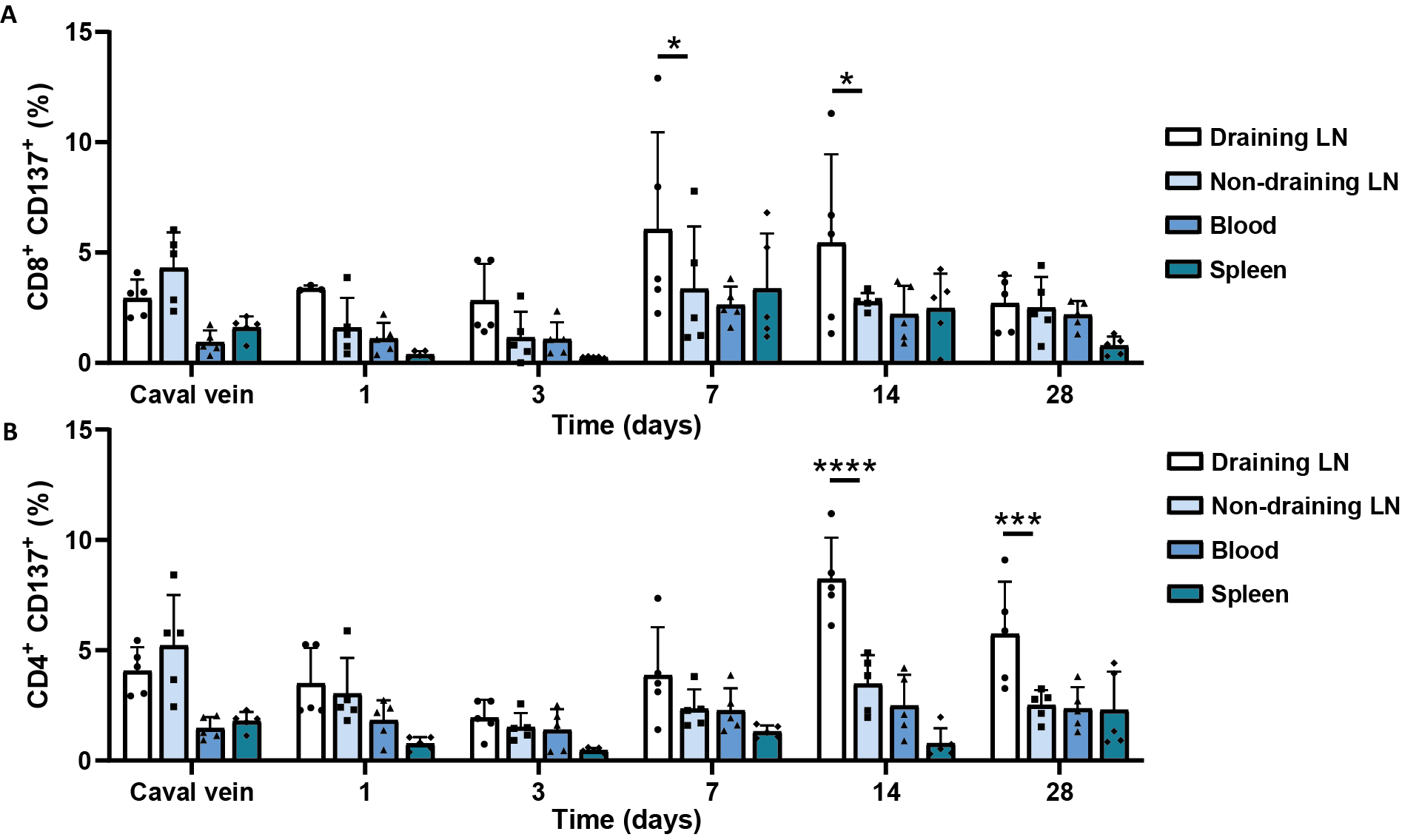


**Figure S4 | Expression of CD137 by CD4^+^ or CD8^+^ T cells in different organs over time.**

Quantification of blood, spleen, draining and non-draining (inguinal) lymph node CD8^+^ CD137^+^ (**A**) and CD4^+^ CD137^+^ (**B**) T cells. VC: vena cava. Data is represented as mean ± SD and were tested using two-way ANOVA with Dunnett post-hoc test, significance is indicated for relevant comparisons, and *p<0.05, **p<0.01, and ***p<0.001.


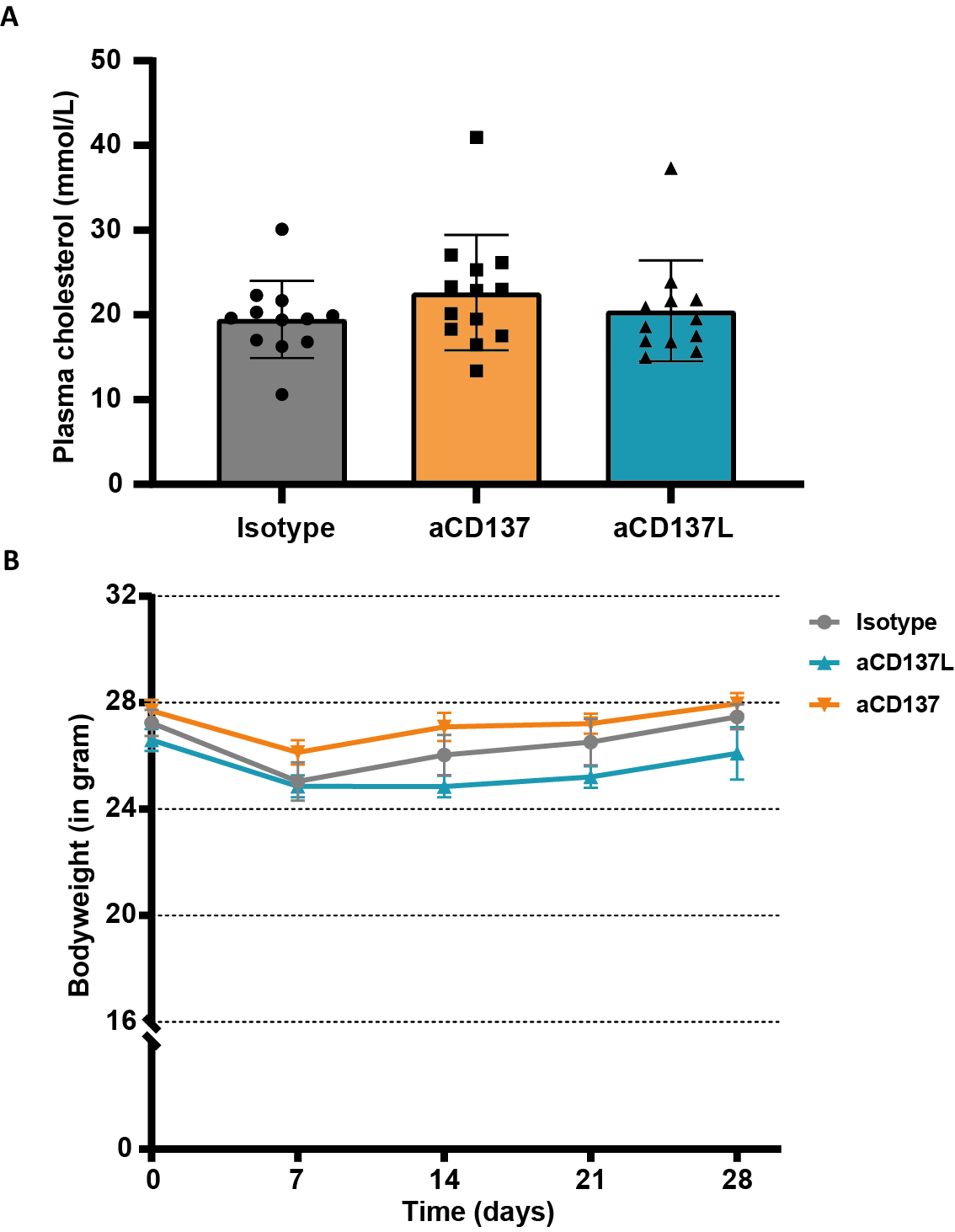


**Figure S5 | Plasma cholesterol of hypercholesterolemic ApoE3*Leiden mice prior to surgery and bodyweight after surgery (n=12-13/group).** Data is represented as mean ± SD and tested using Kruskal-Wallis with Dunn post-hoc test(**A**) or two-way ANOVA with repeated measures and Dunnett post-hoc test(**B**), and *p<0.05, **p<0.01, and ***p<0.001.

**
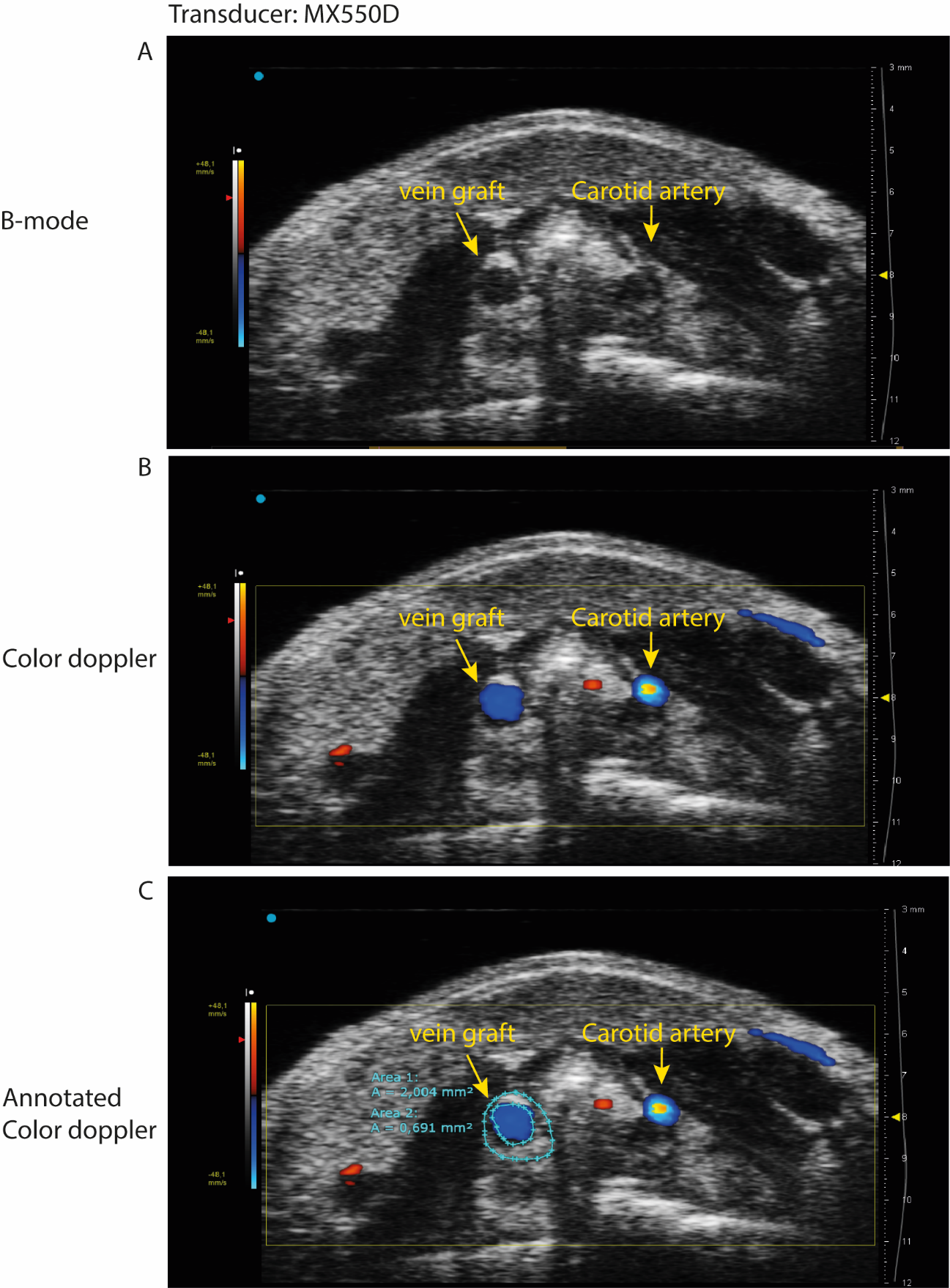
**

**Figure S6 | Ultrasound analysis of vein grafts of ApoE3*Leiden mice.** A 3D B-mode of the neck region of the mouse is acquired using the MX550D transducer. Representative images showing the vein graft (on the left) and the carotid artery (on the right) on B-mode (**A**) and Color doppler (**B**). Manual annotation (**C**) depicts the lumen and vessel wall area.

**
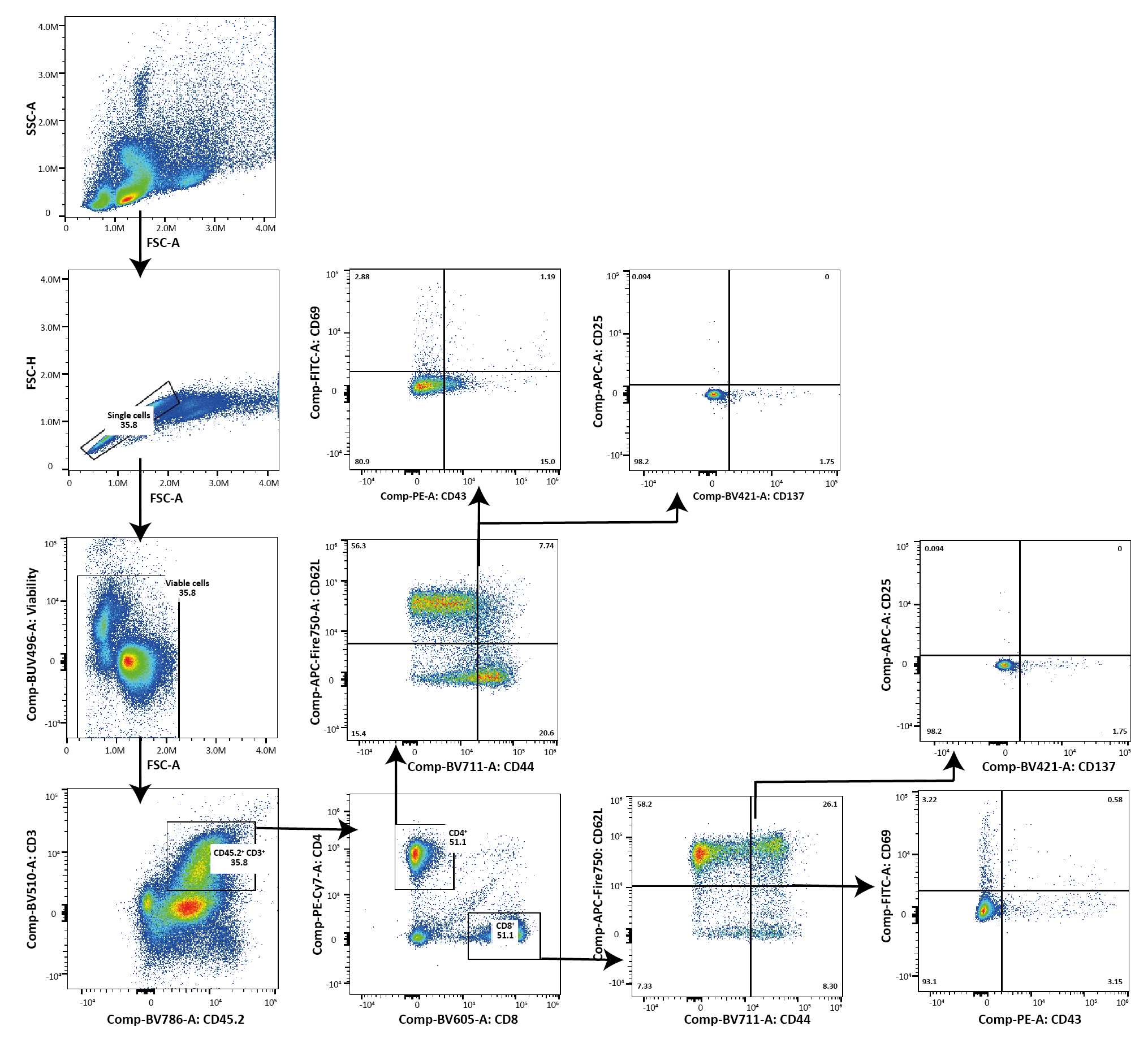
**

**Figure S7 | Flow cytometry gating strategy investigating differentiation of PBMCs following therapeutic targeting of CD137-CD137L costimulatory pathway.** Representative example of gating strategy used to assess CD4^+^ and CD8^+^ T cell polarization in PBMCs following therapeutic targeting of CD137-CD137L costimulatory pathway. At sacrifice, blood was drawn via the orbital sinus and consequently processed for flow cytometric analysis (n=8-9/group).

**
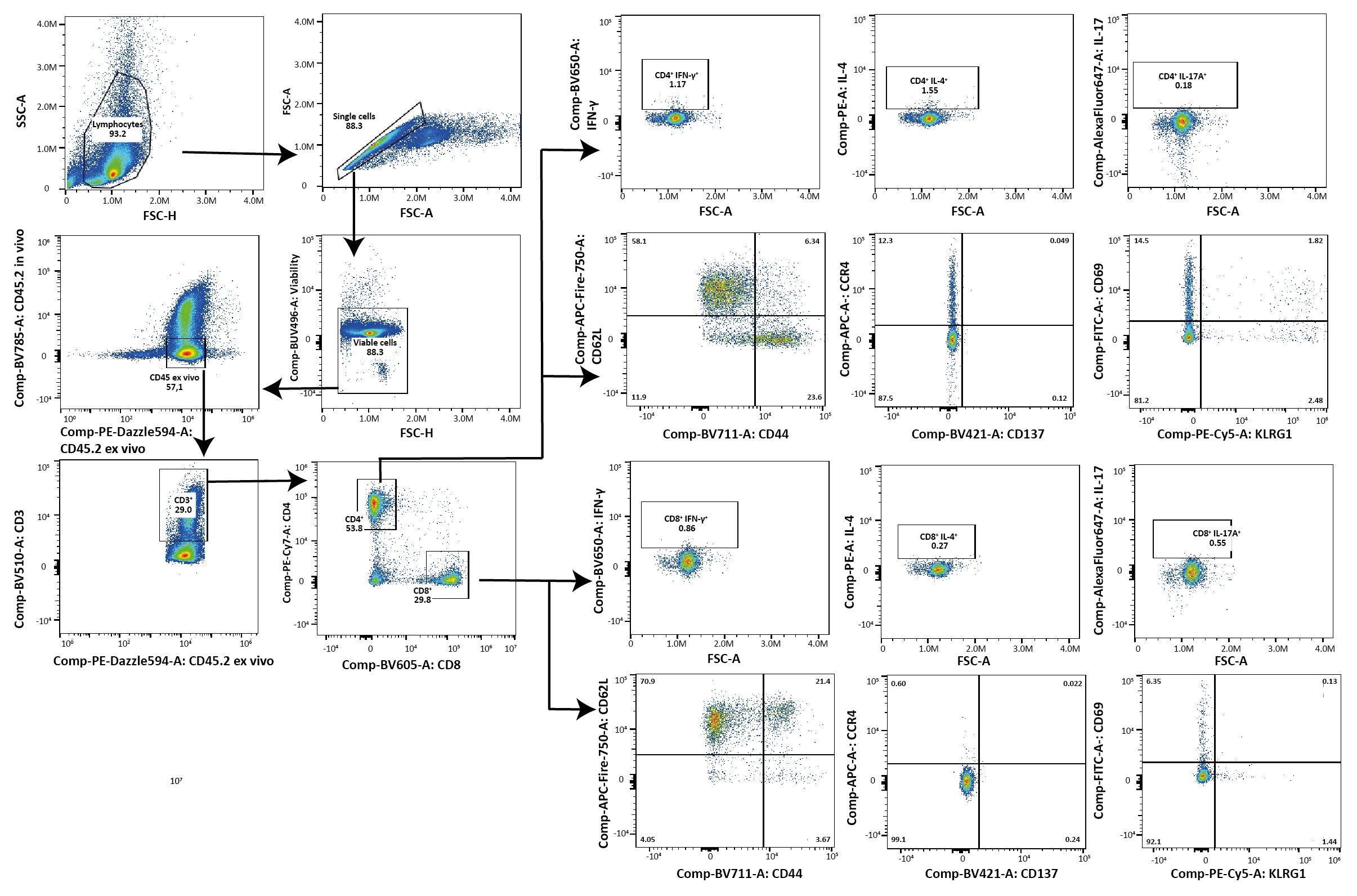
**

**Figure S8 | Flow cytometry gating strategy investigating lesional CD8^+^ and CD4^+^ T cell polarization following therapeutic targeting of CD137-CD137L costimulatory pathway.** Representative example of gating strategy used to assess CD4^+^ and CD8^+^ T cell polarization in murine vein grafts (n=4-6/group) following therapeutic targeting of CD137-CD137L costimulatory pathway. Single cell suspensions from atherosclerotic vein grafts were obtained by enzymatic digestion and consequently stained for flow cytometric analysis.

**
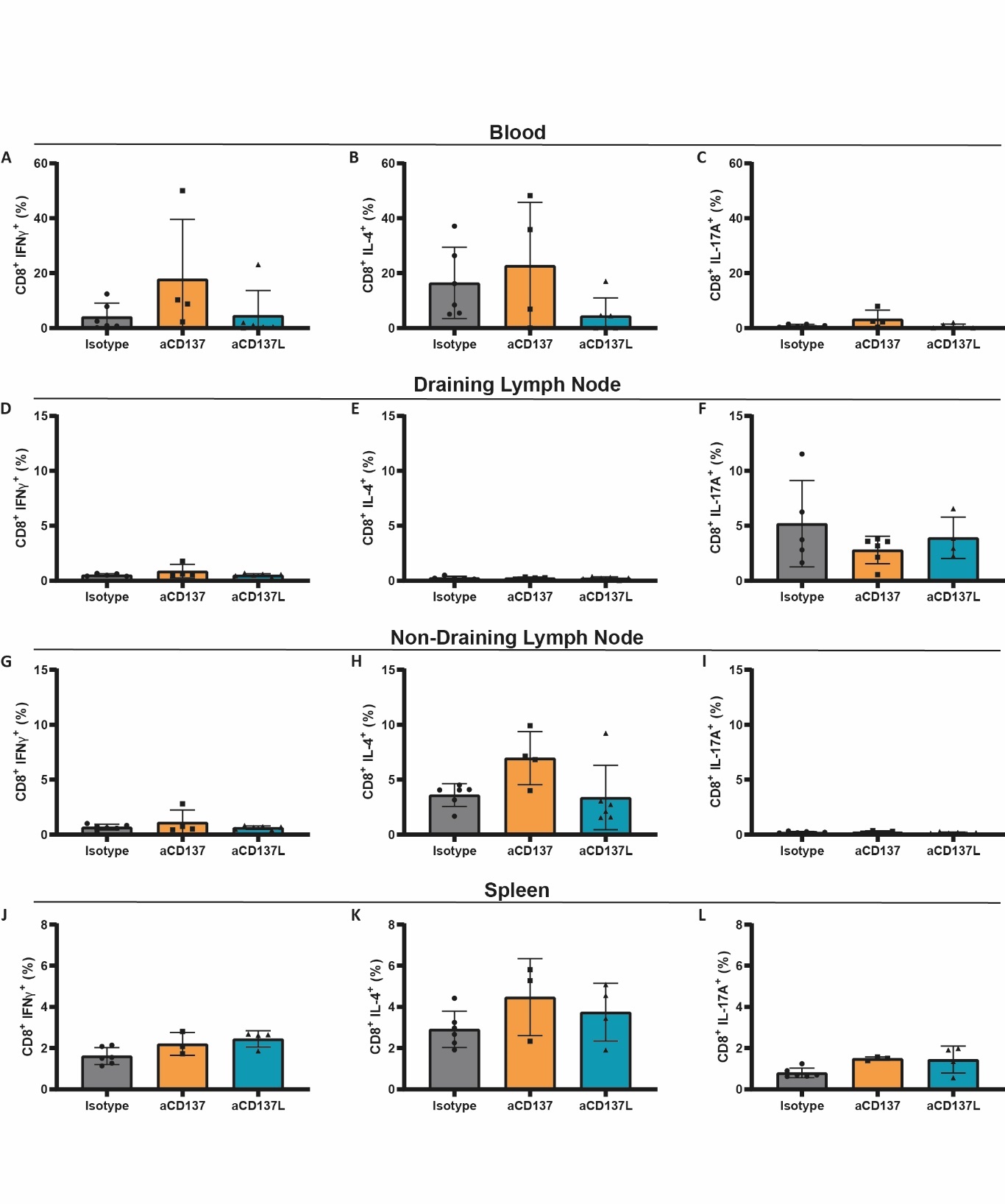
**

**Figure S9 | CD8^+^ T cell polarization in different organs following therapeutic targeting of CD137-CD137L costimulatory pathway.** Expression of IFN-γ, IL-4, and IL-17A by CD8^+^ T cells in blood (**A-C**), draining (**D-F**) and non-draining (**G-I**) lymph nodes as well as spleen (**J-L**) by CD8^+^ T cells after treatment with either isotype, agonistic or antagonistic CD137 antibodies (n=4-6/group). Data is represented as mean ± SD and tested using one-way ANOVA (ANOVA overall not-significant), and *p<0.05, **p<0.01, and ***p<0.001.

**
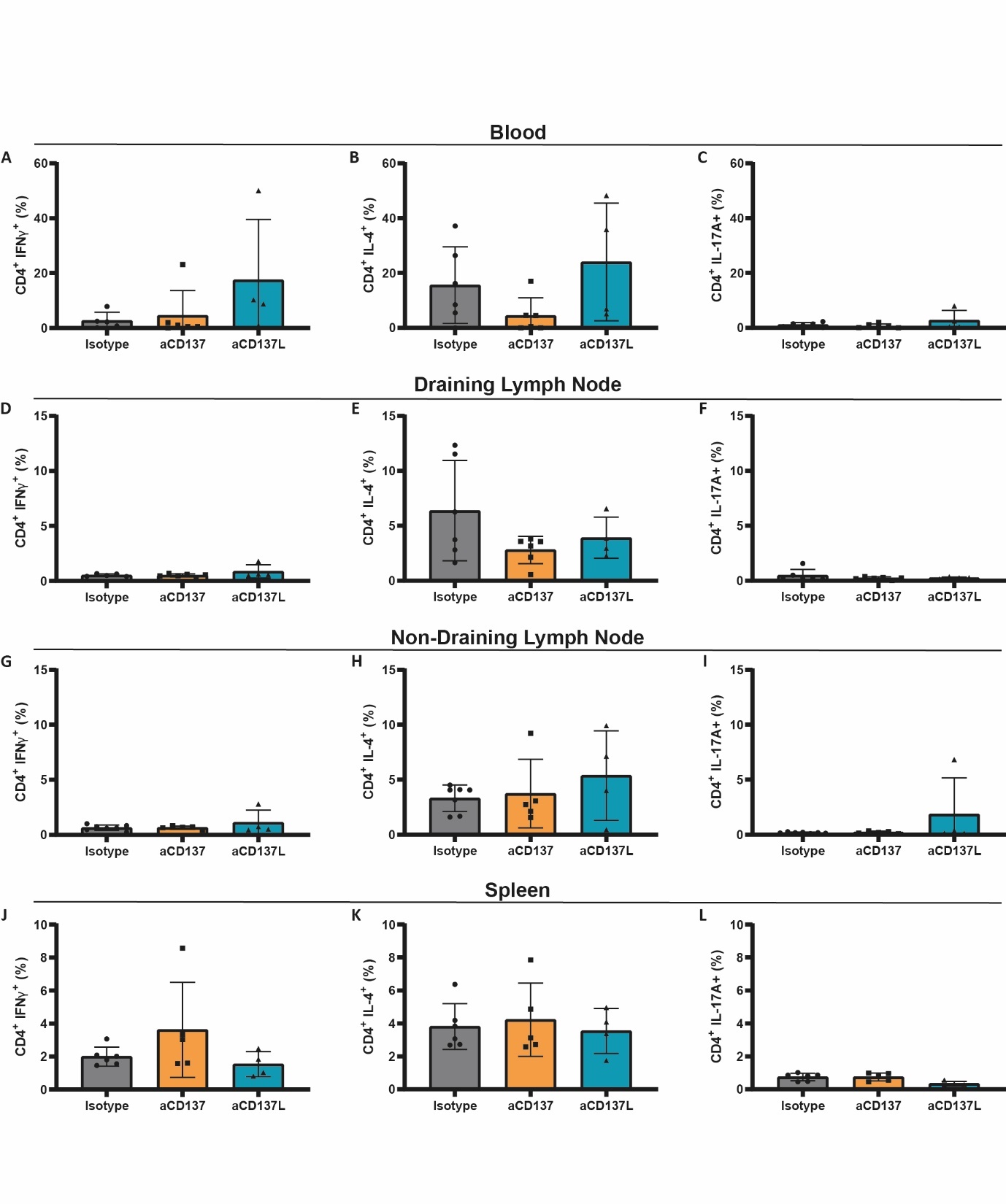
Figure S10 | CD4^+^ T cell polarization in different organs following therapeutic targeting of CD137-CD137L costimulatory pathway.** Expression of IFN-γ, IL-4, and IL-17A by CD8^+^ T cells in blood (**A-C**), draining (**D-F**) and non-draining (**G-I**) lymph nodes as well as spleen (**J-L**) by CD4^+^ T cells after treatment with either isotype, agonistic or antagonistic CD137 antibodies (n=4-6/group). Data is represented as mean ± SD and were tested using one -way ANOVA (ANOVA overall not significant), and *p<0.05, **p<0.01, and ***p<0.001.

**
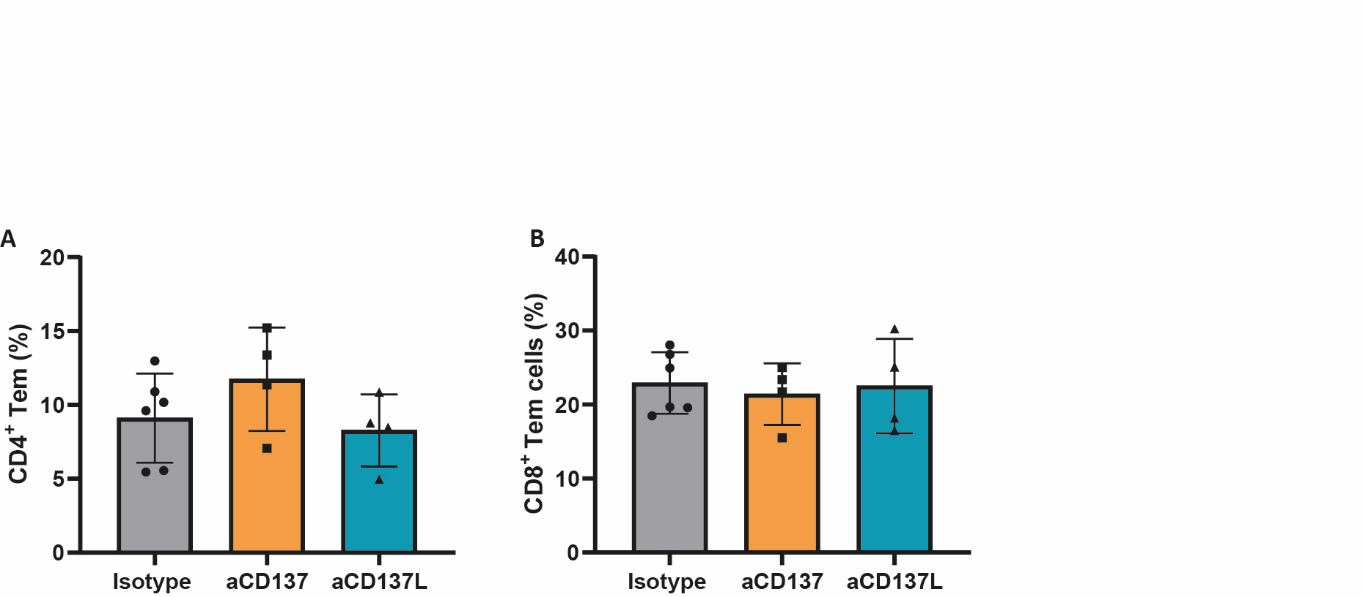
**

**Figure S11 | CD4^+^ and CD8^+^ T cell differentiation in blood following therapeutic targeting of CD137-CD137L costimulatory pathway.** Flow cytometric analysis of CD4^+^ (**A**) and CD8^+^ T cell differentiation (**B**) after treatment with either isotype, agonistic or antagonistic CD137 antibodies (n=4-6/group). Data is represented as mean ± SD were tested using one-way ANOVA (ANOVA overall not significant) and *p<0.05, **p<0.01, and ***p<0.001.
